# Deciphering dengue: novel RNA barcoding segments for enhanced serotype-specific identification and global surveillance of dengue viruses

**DOI:** 10.3389/fmicb.2024.1474406

**Published:** 2024-12-23

**Authors:** Shuai Jiang, Gaili Zhao, Yunyun Ding, Shunxing Ye, Zeqi Li, Changqiao You, Yan Yin, Xinhong Guo

**Affiliations:** ^1^College of Biology, Hunan University, Changsha, China; ^2^College of Bioscience and Biotechnology, Hunan Agricultural University, Changsha, China

**Keywords:** four DENV serotypes, RNA barcoding technology, species-specific molecule markers, population genetic tests, genetic information visualization

## Abstract

**Introduction:**

Dengue viruses (DENVs), the causative agents of dengue hemorrhagic fever and dengue shock syndrome, undergo genetic mutations that result in new strains and lead to ongoing global re-infections.

**Objectives:**

To address the growing complexity of identifying and tracking biological samples, this study screened RNA barcode segments for the four DENV serotypes, ensuring high specificity and recall rates for DENV identification using segments.

**Results:**

Through analyzing complete genome sequences of DENVs, we screened eight barcode segments for DENV, DENV-1, DENV-2, DENV-3, and DENV-4 identification. Comparing the screened barcode segments to sequences of known strains and determining the proportion of correctly or incorrectly identified nucleotides, these segments demonstrated an average recall rate at nucleotide level of 91.34% for four DENV serotypes, a specificity of 99.50% at species level within the *Flaviviridae* family, and a precision rate of 100% for identifying DENVs. For arboviruses, the nucleotide-level specificity was 63.58%. We designed and used the “Barcoding” software to streamline segment design, integrating automated sequence preprocessing, evaluation of barcode segments, and primer design, significantly reducing manual intervention and enhancing overall efficiency. We also established an online database called “Barcodes” for storing and preparing barcode segments.

**Conclusion:**

This work established a standard framework for DENV identification and barcode segment selection, promising significant advancements in the real-time management and control of DENVs, thereby enhancing surveillance capabilities and facilitating targeted interventions in dengue outbreak-prone regions.

## Highlights

• We standardized the framework for RNA barcoding technology and optimized the existing barcode segment validation algorithms.
• Highly conserved barcode segments of DENVs were intercepted through SNP sites.
• The barcode segments achieved a perfect identification precision rate of 100% for DENVs, as validated through Blast analysis against a large dataset of sequences from the GISAID’s EpiCoV, GISAID’s EpiArbo, BV-BRC, NGDC, NCBI-influenza and RVDB databases.
• These barcode segments can effectively and reliably identify DENVs from high-throughput sequencing data.
• The “Barcoding” software streamlined the segment design procedures and increased analytical efficiency.

## Introduction

Four distinct serotypes of dengue viruses (DENVs), DENV-1 to DENV-4, belong to the *Orthoflavivirus* genus within the *Flaviviridae* family (Brillet et al., 2024). These small and single-stranded RNA viruses exhibit genetic differences that allow multiple infections in humans, each compromising specific long-term immunity (Paz-Bailey et al., 2024). DENVs are primarily transmitted through bites from *Aedes aegypti* and *Aedes albopictus* mosquitoes, posing significant public health challenges in tropical and subtropical regions (Porse et al., 2015). Recent advancements in whole-genome sequencing and molecular biology techniques have provided detailed insights into the genetic diversity and evolutionary dynamics of DENV, enabling more precise characterization of viral lineages and transmission patterns (Stica et al., 2022; Boolchandani et al., 2019).

The concept of Barcoding technology, first proposed by Paul Hebert in 2003, has been crucial in biodiversity research and species identification (Pomerantz et al., 2018). By sequencing specific nucleic acid segments, often from chloroplasts or mitochondria, it enables rapid and accurate identification of a specific species. Besides, this technique has been widely applied for virus identification. For instance, Lam et al. (2020) identified SARS-CoV-2-related coronaviruses (SARSr-CoV-2) from pangolin tissues using species-specific markers. Dr. You distinguished SARS-CoV-2 from human coronaviruses (HCoVs) and SARSr-CoV lineages through genomic data analysis (You et al., 2024). The application of barcoding technology appears to offer an efficient molecular tool for DENVs’ identification and detection, as suggested by recent findings.

Real-time quantitative reverse transcription polymerase chain reaction (RT-qPCR) remains the gold standard for DENV detection and serotyping in viremic (RNA-positive) cases, offering high sensitivity and specificity (Álvarez-Díaz et al., 2021). Additionally, serological tests such as enzyme-linked immunosorbent assay and neutralization assays are employed to assess infection rates and vaccine efficacy (e.g., CYD-TDV) (Galula et al., 2021). Advanced methods like amplicon-based sequencing have also been developed, such as the “DengueSeq” protocol (Vogels et al., 2024), enabling cost-effective, full genome recovery of all dengue virus serotypes using multiplexed sequencing on platforms like Nanopore. However, these methods have limitations: they are time-consuming, labor-intensive, and require specialized equipment and trained personnel, which may not be feasible in resource-limited settings. They are also heavily dependent on the timing of sample collection, with late collections potentially leading to decreased viral loads and false-negative results (Kok et al., 2023). Methods like RT-qPCR and next-generation sequencing require high-quality RNA samples collected during a narrow window of viremia. In comparison, barcoding technology provides a rapid and cost-effective alternative for DENV detection. This method significantly reduces detection time compared to RT-qPCR and amplicon-based sequencing, offering results in less than 2 h due to simplified procedures that require less manual handling and no need for thermal cycling equipment or extensive amplification cycles. Barcoding technology is less dependent on the timing of sample collection, as it can detect viral genetic material even when viral loads are low, thereby minimizing false-negative results associated with late sample collection. While barcoding technology may not offer full genome information, it simplifies the detection process by reducing the need for complex data analysis and specialized equipment. This technology also shows potential in identifying genetic variations among DENV strains, which is crucial for epidemiological surveillance and timely public health interventions. Although metagenomic sequencing is required for full genome characterization of new viruses, barcoding technology provides a valuable initial screening tool that enhances the efficiency of dengue surveillance programs. Furthermore, barcode segments can be utilized to design specific probes with fluorescent labels for RT-PCR to enhance serotype identification (Zhuang et al., 2023). By targeting conserved regions unique to each DENV serotype, these barcoded probes improve the specificity and sensitivity of RT-PCR assays. This integration not only accelerates the diagnostic process but also provides detailed serotype information essential for epidemiological surveillance and outbreak management.

Due to their extensive variability and global distribution, DENVs are crucial for informing public health surveillance strategies, such as outbreak prediction, monitoring transmission dynamics, and guiding vaccine deployment efforts (Cattarino et al., 2020). Barcoding technology has improved the rapid detection of microbial samples from environmental matrices, enabling detection within hours, along with precise serotyping and data analysis of DENVs’ genetic diversity and evolutionary trajectories (Johnson et al., 2005). This advancement elucidates intricate genetic relationships among DENV serotypes, providing key insights into their adaptive mechanisms and mutation patterns (Bell et al., 2019). Examining DENV surface protein genes has highlighted viral mutation trends, providing crucial insights into antigenic variability, which are essential for evaluating vaccine efficacy and developing evidence-based intervention strategies (Afroz et al., 2016; Villar et al., 2015; Rather et al., 2017). Barcoding technology based on the high-throughput sequencing results allows rapid and extensive analysis of viral sequences, significantly enhancing the efficiency of identifying multiple viral variants in a single run, which is essential for broad DENV surveillance during epidemic seasons and early outbreak characterization (Massart et al., 2019). Comprehensive phylogenetic and mutation analysis of DENV genetic data enables researchers to explore mechanisms of host and environmental adaptation. In terms of host adaptation, this includes mutations that affect immune evasion and receptor binding affinity (Zimmerman et al., 2018). Regarding environmental adaptation, it involves alterations in viral replication efficiency at different geographical locations or changes in vector transmission efficiency, which have led to the spread of DENVs across various ecological niches. These findings have provided detailed insights into how DENVs evolve to maintain infectivity and transmissibility, explaining their resilience and persistence in both endemic and newly affected regions.

Leveraging design principles from SARS-CoV-2 RNA barcoding studies, we refined RNA barcoding methodologies applied on DENVs by optimizing the algorithms for evaluating barcode segments and performing primer simulation, design, and validation (Lam et al., 2020; You et al., 2024). We aimed to create barcode segments for both interserotype (DENV strain-specific) and intraserotype (four DENV serotypes-specific) identification, evaluating their accuracy and applicability for identifying cryptic viral specimens. Using multiple sequence alignment of the four DENV serotypes, we developed a training set (TRS) to assess genetic diversity and elucidate phylogenetic relationships. Single nucleotide polymorphism (SNP) sites for each DENV serotype were identified and used to segment initial sequences. High-quality barcode segments were selected using the basic local alignment search tool (Blast) and evaluated with a suite of testing evaluation sets (TESs) for testing the ability of barcode segments to identify four DENV serotypes across different taxonomic groups. These segments, along with ancillary data, were visualized in one- (1D) and two-dimensional (2D) codes and made accessible through an online database.^1^ Researchers can access the detailed barcode segments via 2D code scanning or by directly visiting the online database. Additionally, the “Barcoding” Windows client software automated the batch processing of experimental data by incorporating functions (such as data importation, sequence preprocessing, the evaluation of the identification capability of barcode segments, the visualization of barcode segments and the simulation of the primer design for barcode segments) into a unified pipeline, thereby enhancing the efficiency of viral identification processes.

## Materials and methods

**FIGURE 1:**
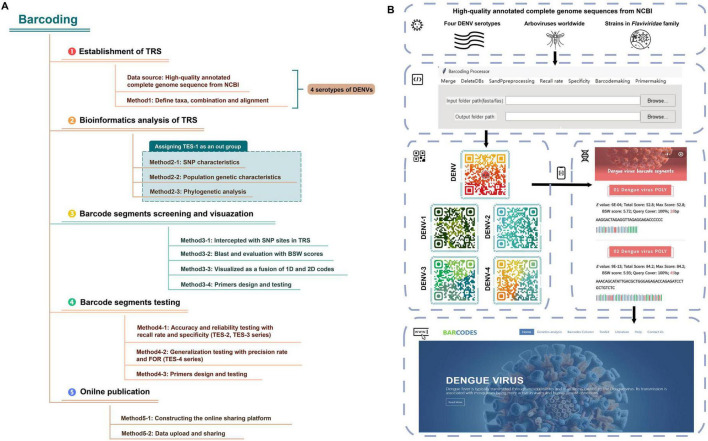
The flowchart of RNA barcoding. **(A)** The technology roadmap of RNA barcode technology. **(B)** The operational flowchart of RNA barcode technology.

The technology roadmap and operational flowchart of RNA barcode technology were shown in Figures 1A, B. Given the widespread prevalence of dengue in many tropical countries, effective surveillance and rapid identification methods were crucial for controlling outbreaks (World Health Organization, 2023)^2^. High notification rates (≥100 per 100,000 persons) were observed in some Latin American countries, including Mexico, Brazil and Colombia, as well as in Southeast Asia (e.g., The Philippines et al.). Moderate rates (1.0–99 per 100,000 persons) were reported in countries like Sudan, Pakistan, Indonesia, India and Australia, whereas lower rates (0.01–0.99 per 100,000 persons) were noted in countries like China and the United States.

### Establishment and bioinformatic analysis of TRS

The complete genomic sequences of the four DENV serotypes (DENV-1 to DENV-4) were curated from the NCBI^3^ (Lam et al., 2020). This compilation served as the foundation for the TRS, with core sequence metrics tabulated in Table 1 and search details in Supplementary Table 1, sheets 1–4. Sequences with significant length variation were removed, resulting in datasets containing: 2,157 sequences of DENV-1 (average length 10,647bp), 1,813 sequences of DENV-2 (average length 10,656bp), 1,003 sequences of DENV-3 (average length 10,650bp) and 257 sequences of DENV-1 (average length 10,616 bp). To enhance alignment precision and facilitate integration with bioinformatic tools, all TRS sequences underwent rigorous refinement using the custom software “Barcoding” (a detailed description of “Barcoding” was provided in the section “Development of barcode-assisted design software”). This involved excising degenerate bases (R, Y, M, K, S, W, H, B, V, D, N) and standardizing sequence lengths to ensure consistency across the dataset. To elucidate the genetic diversity of DENVs and assess barcode segment identification capabilities, we compiled all complete RefSeq genome sequences of all *Flaviviridae* species from NCBI, encompassing five genera: *Flavivirus*, *Hepacivirus*, *Orthoflavivirus*, *Pegivirus*, *Pestivirus* [basic information of TES-1 and detailed annotations about these sequences including accession and version numbers, names of strains and sequence types were provided in Table 2 and Supplementary Table 2, sheet 1, based on NCBI-taxonomy^4^ and the International Committee on Taxonomy of Viruses^5^ (Federhen, 2012; Schoch et al., 2020; Siddell et al., 2023)]. These sequences formed TES-1, which included 651 sequences providing a comprehensive reference for comparative analysis and ensuring that our evaluation considered the full spectrum of genetic variability within the *Flaviviridae* family.

**TABLE 1:** Basic information of TRS (the four DENV serotypes).

Serotypes	Accession and version numbers on NCBI	Number of sequences	Average complete genome length (bp)	Segment length (bp)	Collection date
DENV-1	Details in Supplementary Table 1	2,157	10,647	Details in Supplementary Table 1	Details in Supplementary Table 1
DENV-2	Details in Supplementary Table 1	1,813	10,656	Details in Supplementary Table 1	Details in Supplementary Table 1
DENV-3	Details in Supplementary Table 1	1,003	10,650	Details in Supplementary Table 1	Details in Supplementary Table 1
DENV-4	Details in Supplementary Table 1	257	10,616	Details in Supplementary Table 1	Details in Supplementary Table 1

**TABLE 2:** Basic information of TES-1 (*Flaviviridae* family).

Genera	Accession and version numbers	Taxa	Number of sequences	Average complete genome length	Segment length (bp)
*Flaviviridae*	Details Supplementary Table 2	Taxon1-5	651	10,401	Details Supplementary Table 2
*Flavivirus*	Details Supplementary Table 2	Taxon1	76	11,801	Details Supplementary Table 2
*Hepacivirus*	Details Supplementary Table 2	Taxon2	291	9,449	Details Supplementary Table 2
*Orthoflavivirus*	Details Supplementary Table 2	Taxon3	153	10,649	Details Supplementary Table 2
*Pegivirus*	Details Supplementary Table 2	Taxon4	52	9,993	Details Supplementary Table 2
*Pestivirus*	Details Supplementary Table 2	Taxon5	79	12,344	Details Supplementary Table 2

Given the minor length variations among TRS sequences, we employed MAFFT v7 (online version^6^) for global alignment (Rozewicki et al., 2019). Subsequent analyses with MEGA v11 software delineated SNP characteristics (Tamura et al., 2021), identified base substitutions, and calculated genetic evolutionary distance indices (GEDI) matrices using the Kimura 2-parameter model to assess genetic divergence for inter- and intra-taxa within TRS and TES-1. These matrices were visualized using the heatmap format in R language (ggplot2 package v3.5.1, algorithm: Euclidean distance with average linkage clustering) (Zhao et al., 2014; Wang et al., 2023). Additionally, “Gene Flow and Genetic Differentiation” analyses on TRS and TES-1 were performed using DnaSP v6 (Rozas et al., 2017). These analyses provided insights into population genetic differences, evolutionary processes, and speciation mechanisms among DENVs and within the *Flaviviridae* species (Waman et al., 2016).

### Construction of phylogenetic trees

To improve the accuracy of phylogenetic analysis and reduce computational load, we utilized a curated dataset of high-quality DENV serotype sequences from the taxonomy database.^7^ This dataset comprising 3 DENV-1, 2 DENV-2, 5 DENV-3, and 5 DENV-4 sequences was used to replace the TRS and subsequently merged with TES-1 to establish a phylogenetic tree reflective of the taxonomic levels pertinent to DENVs. The accession and version numbers of these sequences (listed in Supplementary Table 1, sheet 5) were provided to allow for verification of the exact sequences used and to facilitate reproducibility in future research. The function of measuring substitution saturation in the DAMBE v7.3.32 software was used to evaluate the obtained tree construction reliability (Xia, 2018). The feature of “Find Best DNA/Protein” models and the optimal substitution model in MEGA v11 were used to fabricate the tree based on the maximum likelihood algorithm (the bootstrap value of the maximum likelihood tree was set to 90% with 1,000 replicates to ensure the stability and reliability of the results). Additionally, an intraspecific bayesian evolutionary tree for DENVs at the species level predicated on the Markov chain Monte Carlo algorithm was constructed using BEAST v1.10 software to validate the phylogenetic outcomes (the posterior probability of the bayesian tree was set to 90% to ensure the stability and reliability of the results) (Suchard et al., 2018). The obtained phylogenetic tree was visualized online via iTOL v6 (Letunic and Bork, 2024).^8^

### Barcode segments screening and visualization

Through DnaSP v6, SNP sites for the entirety of DENV as well as for each of the four serotypes within TRS were identified (Page et al., 2016). Using these SNP sites as boundaries, we partitioned the sequences to obtain the original segments. The retained segments were aligned against the standard nucleotide database within the NCBI Blast portal^9^ (Pruitt et al., 2005), encompassing all nucleotide sequences, totaling approximately 107 million sequences across all species as of July 20, 2024. The “Program selection parameters” (the target identity was 95%) and “Max target sequences” (selected the maximum number of aligned sequences to display) were adjusted to the highest value (5,000 comparison outcomes) to enhance the reliability of the Blast results. When all 5,000 outcomes belonging to the same DENV serotype showed a “Percent Identity” of 100%, the segments were deemed capable of accurately and reliably identifying a specific serotype. This consistent 100% identify across all comparisons provided empirical evidence qualifying them as serotype-specific barcode segments for DENVs.

Some Blast outcomes were categorized as “partial genome/cds,” leading to scenarios where a uniform “Percent Identity of 100%” in coverage rates for some segments was not obtained. In Blast alignment results, longer sequence alignments typically produced higher total scores (Altschul et al., 1997). This observation was significant for our results because, when identifying DENV sequences using barcode segments, longer alignments could enhance the confidence in matching accuracy. However, the total score was not solely dependent on length; it was also influenced by the number of matched bases, mismatches, and insertions or deletions. These factors could affect the accuracy of viral identification, highlighting the need to consider both alignment length and sequence quality in our analyses. Leveraging the “Conserved Regions” functionality in DnaSP v6, which detected sequence regions that remained unchanged (highly conserved) across multiple sequences, we set the minimum window length to 20 nucleotides and the conservation threshold to 100%. We chose a minimum window length of 20 nucleotides to ensure that the conserved regions were sufficiently long for reliable analysis, as shorter regions might not provide meaningful conservation data. A conservation threshold of 100% was selected to focus exclusively on regions that were completely conserved across all sequences in the dataset. This approach allowed us to calculate conservation test scores (*P*-values) for the barcode segments extracted from the TRS, thereby assessing their conservation levels (Riaz et al., 2011). Marrying the strengths of Blast alignment and the DnaSP v6 algorithm, we introduced a new metric called the barcode segment weight (BSW) score, defined by the equation: BSW = lg(max total Blast score/*P*-value). In this equation: Max total Blast score referred to the highest total alignment score obtained from Blast searches for a given barcode segment. This score reflected the degree of similarity between the barcode segment and reference sequences in the database; higher scores indicated greater similarity. *P*-value was the conservation test score calculated by DnaSP v6 for the barcode segment. It represented the statistical significance of the conserved regions within the segment, with lower *P*-values indicating higher conservation across the sequences analyzed.

All barcode segments were visualized as a combination of the 1D and 2D codes. The 1D codes allowed for direct inspection and analysis of the genetic sequences by researchers, while the 2D codes facilitated rapid and accurate data retrieval through scanning technologies. This combination significantly improved data sharing, storage, and processing efficiency in viral identification workflows. In the 1D representation, nucleotide bases A, T(U), C, and G were encoded in light purple, red, green, and blue, respectively. These colors were chosen for their high contrast and to facilitate easy recognition of each nucleotide during analysis (Li et al., 2021). Additionally, complementary base pairs were visually differentiated using distinct graphical markers to represent their bonding characteristics. The A-T(U) pairs were indicated by long vertical lines, while G-C pairs were represented by short vertical lines. The 2D codes generated through QR Code Generator, encapsulated both the 1D visual representations and essential information of the barcode segments within a dynamic 2D code framework.^10^ Accessing these codes via mobile devices allowed seamless retrieval of information, supported by an error tolerance of 30% (You et al., 2024), ensuring accessibility even with partial image loss.

Finally, primers were meticulously designed for all barcode segments, with amplification fidelity confirmed through the primer-Blast service provided by NCBI^11^ (Ye et al., 2012). Specifically, we evaluated the primers for specificity by ensuring they uniquely matched the target species without significant homology to non-target species. We assessed their melting temperatures to ensure compatibility with PCR conditions. Additionally, we checked for the absence of secondary structures such as hairpins and primer-dimers using Primer-BLAST’s analysis tools. This thorough *in silico* validation ensured that the primers would amplify the intended barcode segments accurately and efficiently.

### Identification capabilities of barcode segments

By July 20, 2024, we had extracted 5,370 full-genome sequences of the four DENV serotypes from NCBI to serve as TES-2 (collection date and search details on NCBI were detailed in Supplementary Table 2, sheet 2). This compilation allowed for cross-validation of the average recall rate of the barcode segments at nucleotide-level and species-level and the recall rate among DENV serotypes at nucleotide-level and species-level.

**FIGURE 2:**
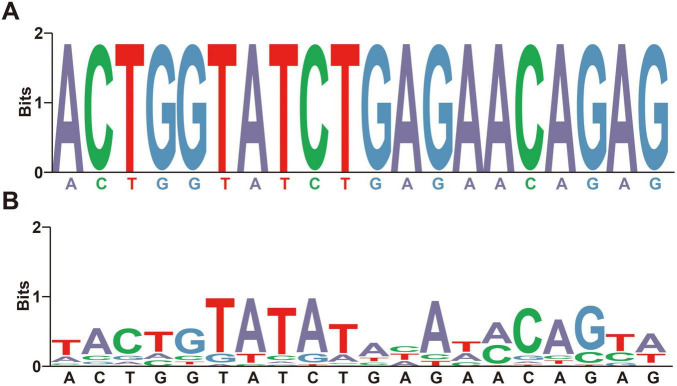
The visualization of recall rate and specificity algorithms. **(A)** The visualization of recall rate algorithms. The identical base colors above and below the horizontal axis indicate a high degree of consistency in nucleotide composition and sequence alignment between the barcode segments and the tested species. **(B)** The visualization of specificity algorithms. The different base colors above and below the horizontal axis indicate a high degree of difference in nucleotide composition or sequence alignment between the barcode segments and the tested species.

To calculate the average nucleotide-level recall rate of the barcode segments, we first aligned each barcode segment with the corresponding regions of all DENV species sequences. For each species, we counted the number of nucleotide sites where the barcode segment and the species sequence matched exactly. We then divided the total number of matching sites across all species by the product of the barcode segment length and the number of species to obtain the average nucleotide-level recall rate: (Σ_*species*_ Number of matching sites)/(Barcode length × Number of species). The average nucleotide-level recall rate was visualized in Figure 2A. We set a threshold to determine the effective recall of the barcode segments for each species. If the nucleotide-level recall rate for a species exceeded the threshold (e.g., 95%), the recall rate for that species was set to 100%; otherwise, it was set to 0%. We then summed these rates across all species and divided by the total number of species: (Σ_*species*_ the average nucleotide-level recall rate)/(Number of species). This binary evaluation simplified the assessment of the barcode’s effectiveness at the species level. The choice of threshold was based on the desired stringency of matching; a higher threshold ensured that only highly similar sequences were considered matches, reducing the possibility of false positives.

To evaluate the specificity of the barcode segments, we aligned them with all non- DENV *Flaviviridae* strains (TES-1) and the complete genomic sequences of globally impactful arboviruses from the NCBI database (Marceau et al., 2016), constructing TES-3-1 and TES-3-2 (names of strains containing in TESs were detailed in Supplementary Table 2, sheets 3, 4). We calculated the nucleotide-level specificity by counting the number of nucleotide mismatches between each barcode segment and the sequences of non-DENV species. Simliarly, the average specificity at the nucleotide and species level was calculated as: (Σ_*species*_ Number of mismatching sites)/(Barcode length × Number of species); (Σ_*species*_ the average nucleotide-level specificity)/(Number of species), respectively. We set a specificity threshold based on the acceptable number of mismatched bases (e.g., if there are more than × different bases between a species and a barcode segment, specificity was set to 100%; otherwise, it was set to 0%). This threshold ensured that only sequences sufficiently different from DENV were considered non-specific, thereby reducing false positives in detection. The visualization method for nucleotide specificity at the average species level was shown in Figure 2B. Besides, for gaps (“-”) existing in sequences after alignment in TES-3-1 and TES-3-2, we treated them as a unique type of base distinct from the standard DNA bases (A, G, C, T) to accurately account for insertions or deletions.

The recall outcomes from aligning barcode segments against species within TESs were significantly impacted by alignment algorithms that process variable regions within the sequences, such as insertions and deletions (indels). This processing led to extensive missing data and gaps, which significantly reduced recall rates and hindered accurate predictions for species with close phylogenetic relationships. For TESs derived from large datasets with abundant internal sequence polymorphism, increasing the threshold value, which set a higher similarity requirement for sequence alignment, paradoxically lowered recall rates because it excluded true positive matches that had natural sequence variations. Conversely, for TESs derived from smaller samples with sample sizes of fewer than 100 sequences and minimal sequence polymorphism, setting the maximum threshold directly might be feasible for calculating recall rates in identifying phylogenetically related species. As the nucleotide divergence among the DENV serotypes was markedly smaller than among the distinct subtypes of the influenza virus or *Flaviviridae* species (Kumari et al., 2023; Rijnink et al., 2023), the thresholds for recall rates were maintained at a comparatively elevated level.

TES-2, TES-3-1, and TES-3-2 were amalgamated with all barcode segments for subsequent comparison. These TESs then utilized “Barcoding” software to evaluate the recall rates and specificity of barcode segments. To investigate the impact of gaps on the diagnostic power of barcode segments, we assessed the presence and extent of gaps within virus sequences covered by barcode segments in the TESs. The measurement of gaps was based on their length relative to the average length of all barcode segments: no gaps (0 bp), small gaps (up to 6 bp, 20% of the average segment length), medium gaps (up to 15 bp, 50% of the average segment length), and large gaps (over 15 bp).

### The generalization testing of barcode segments

The high degree of sequence similarity inherent within the TRS, stemming from conserved genetic regions among the four DENV serotypes, could potentially lead to overfitting. This meant that the barcode segments perform exceptionally well on the TRS data but may not generalize effectively to new, unseen viral sequences when distinguishing the four DENV serotypes. To further assess the identification capabilities of these segments, we constructed TES-4 series to test them against datasets containing viruses of unknown homology or phylogenetically distant strains, such as influenza viruses, coronaviruses, and poxviruses. We utilized the Blast functionalities of several databases to evaluate the generalizability (applicability and consistency) of barcode segment identification across diverse viral species. These databases included GISAID’s EpiCoV^12^ (Torrens-Fontanals et al., 2022), GISAID’s EpiArbo (see text footnote 12), BV-BRC (reference and representative viral genomes)^13^ (Olson et al., 2023), NGDC (covering the *Coronaviridae* and *Poxviridae* families, Monkeypox virus genomes, and NCBI RefSeq viruses representation genomes)^14^ (CNCB-NGDC Members and Partners, 2023), the NCBI-influenza virus resource (Influenza A, B, and C viruses)^15^ (Bao et al., 2008), and RVDB (nucleotide database)^16^ (Goodacre et al., 2018). Parameters for Blast comparisons were uniformly set to “Optimize for highly similar sequences.”

### Development of barcode-assisted design software

We encapsulated Python code into a Windows-compatible software named “Barcoding” (refer to Figure 1B for the software interface and for the software and the user manual/operation guide).^17^ This automation facilitated tasks such as merging sequences, handling degenerate bases, calculating specificity and recall rates, visualizing 1D barcode segments, and designing primers, significantly reducing the development cycle for barcode segments. “Barcoding” was intricately designed for data management and bioinformatics analyses specific to barcoding technology. Emphasizing the Liskov substitution principle (Friedel et al., 2014), the tool was designed with a modular architecture, allowing different components of the code function independently. This modularity enhanced reusability, allowing code components to be used in multiple parts of the program or in other projects without modification.

The graphical user interface offered an intuitive display of information, with straightforward and consistent operation, easing the learning curve for new users. The Barcoding software optimized data batch processing workflow, significantly enhancing the accuracy and efficiency of data analysis compared to manual operations. For instance, a data analysis task that previously required several days of manual work could now be completed in just a few minutes using the software.

### Constructing an online sharing platform

Using a web-based platform and a database management system (Supplementary Table 3), we developed an online database as a service (DBaaS) of DENV barcode segments (Agosto-Arroyo et al., 2017; Oh et al., 2015). This service was accessible via the domain (see text footnote 1) (the main interface of the webpage was shown in Figure 1B). Users could access basic information about barcode segments, download construction codes and software, and obtain genomic annotations for viruses, as well as receive updates on dengue fever outbreaks and scholarly articles from both domestic and international sources through an online platform. Additionally, we have designed an online Blast tool for comparing user-submitted sequences against the DENV barcode segments. Embedded within the Blast tool were barcode segments for DENVs at both species and serotype levels. By adjusting the “Percent identity” parameter, users could fine-tune the balance between sensitivity and specificity in their search results. Lowering the “Percent identity” threshold allowed detection of more distantly related sequences (increasing sensitivity); while raising it yielded more exact matches (increasing specificity). This feature enabled users to obtain comparison results tailored to their specific research needs.

## Results

### SNP analysis

**FIGURE 3:**
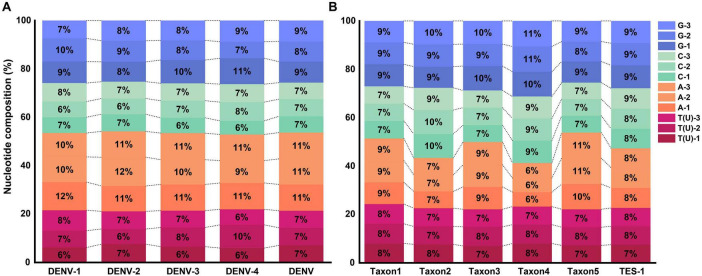
Nucleotide composition at different codon positions. **(A)** Nucleotide composition of TRS. **(B)** Nucleotide composition of TES-1.

The average GC base pair content (BPC) for the four DENV serotypes was 46.3 ± 0.8%, while the AT(U) BPC was 53.7 ± 0.8% (Figure 3A and Supplementary Table 4, sheet 1, which listed the GC and AT(U) content for each serotype in detail). Compared to HCoVs (GC BPC ranging from 30 to 40%) (You et al., 2024), DENVs showed higher genetic stability. The higher GC BPC predisposed DENVs to CpG methylation, a process where methyl groups were added to cytosine nucleotides adjacent to guanine nucleotides (CpG sites), potentially affecting viral gene expression and replication. This underscored their greater genomic stability and provided insights into their molecular evolution and potential mechanisms for epigenetic regulation. Conversely, TES-1 did not exhibit a significant preference for varied bases at identical codon positions or identical bases at various codon positions, though marked disparities in GC BPC (52.6 ± 6.5%) were evident among the taxa, ranging from 46.1% in taxon5 to 58.6% in taxon4. These disparities in GC content could influence the viruses’ mutational biases and adaptability, with taxon1 (48.5%), taxon3 (50.1%) and taxon5 (46.1%) being more prone to adaptive mutations and recombination with phylogenetically proximate strains due to selective pressures (Figure 3B and Supplementary Table 4, sheet 1) (Bobay and Ochman, 2017). Furthermore, there was a notable disparity in overall GC BPC between DENV (46.3%) and taxon3 (50.1%). Specific molecular-level nucleotide genetic markers significantly facilitated distinguishing DENVs from closely related species within the *Flaviviridae* family and even those in the *Orthoflavivirus* genus.

**FIGURE 4:**
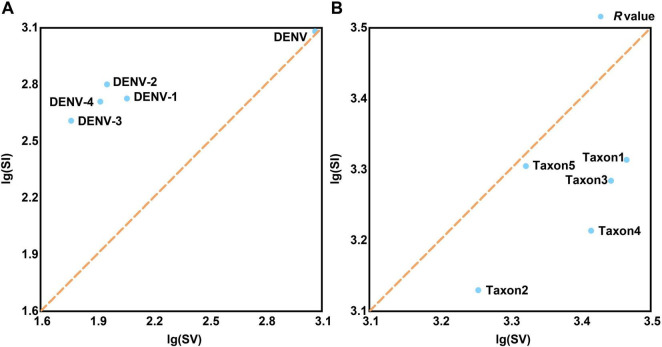
The tests for the frequencies of base substitution. **(A)** The frequencies of base substitution in TRS. **(B)** The frequencies of base substitution in TES-1. The common logarithmic treatment is applied since the SI and SV values of viral strains differ significantly. The brown dashed diagonal line (x = y) divides the coordinate system into upper and lower regions. The *R*-value [the ratio of lg(SI) and lg(SV)] anchor point is above the line (*R*-value > 1), suggesting that the species’ base substitution form is biased toward SI, otherwise the form is biased toward SV (*R*-value < 1). SI, transitional pairs; SV, transversional pairs.

Base substitution analysis revealed that all four DENV serotypes exhibited *R*-values above 1, indicating a predominance of transitionsal pairs (SI) without notable extremes (Figure 4A and Supplementary Table 4, sheet 2). This finding suggested low nucleotide substitution saturation, meaning that the rate of mutation accumulation has not reached a level where it obscured true evolutionary signals. Additionally, the analysis indicated reduced evolutionary noise, referring to the limited interference from random mutations, and robust evolutionary propulsion, indicating strong directional selection maintaining specific mutations. These factors might contribute to the persistent recurrence of dengue outbreaks by ensuring that advantageous mutations are conserved and promoted through natural selection (Schmidt et al., 2011). In contrast, base substitution dynamics across the serotypes were primarily characterized by transversional pairs (SV) with *R*-values below 1, notably with taxon5 registering the highest *R*-value at 0.99 (Figure 4B and Supplementary Table 4, sheet 2). *R*-values below 1 indicated a higher rate of transversion mutations relative to transitions, which could lead to more radical changes in the nucleotide sequence. These findings indicated that while most *Flaviviridae* species, such as West Nile virus and Zika virus, maintained stable evolutionary trajectories characterized by balanced mutation rates; viruses with multiple serotypes or subtypes, such as DENVs and Hepatitis C virus, possessed broader evolutionary capacities. These evolutionary capacities include a higher propensity for recombination with rapidly evolving species, such as influenza viruses or SARS-CoV-2. This increased recombination potential necessitated stricter conservation of molecular genetic markers to ensure accurate DENV identification (Focosi and Maggi, 2022). Additionally, the significant share of identical pairs among the four DENV serotypes (>93%, Supplementary Table 4, sheet 2) provided a wide range of sequence variations for barcode segment extraction. This extensive sequence space (identical pairs) allowed for the development of highly specific and sensitive barcode segments that could effectively distinguish between closely related viral strains.

Substitution saturation testing results (Table 3) (*P*-value < 0.0185) showed that Iss values were consistently lower than the Iss.cSym values across all categorized groups. The Iss measured the extent to which multiple substitutions have occurred at the same nucleotide site, potentially obscuring true evolutionary relationships. An Iss value lower than Iss.cSym indicated that substitution saturation was minimal, ensuring that the sequence data retained sufficient phylogenetic signal for reliable evolutionary analysis. For the *Flavivirus* species, the Iss and Iss.cSym values were closely aligned (0.7370 vs 0.7895). This proximity suggested that substitution saturation was approaching a level that could compromise the accuracy of phylogenetic inferences within the *Flaviviridae* family.

**TABLE 3:** The substitution saturation testing results of TRS and TES-1.

Species	Taxa	Iss	Iss.cSym	*P*-value
DENVs	TRS	0.3126	0.8348	0.0000
*Flavivirus*	Taxon1 in TES-1	0.7370	0.7895	0.0004
*Hepacivirus*	Taxon2 in TES-1	0.3925	0.8228	0.0000
*Orthoflavivirus*	Taxon3 in TES-1	0.6083	0.8408	0.0000
*Pegivirus*	Taxon4 in TES-1	0.6138	0.8335	0.0000
*Pestivirus*	Taxon5 in TES-1	0.4973	0.8400	0.0000
*Flaviviridae*	TES-1	0.5798	0.7218	0.0185

### Population genetic characteristics

**FIGURE 5:**
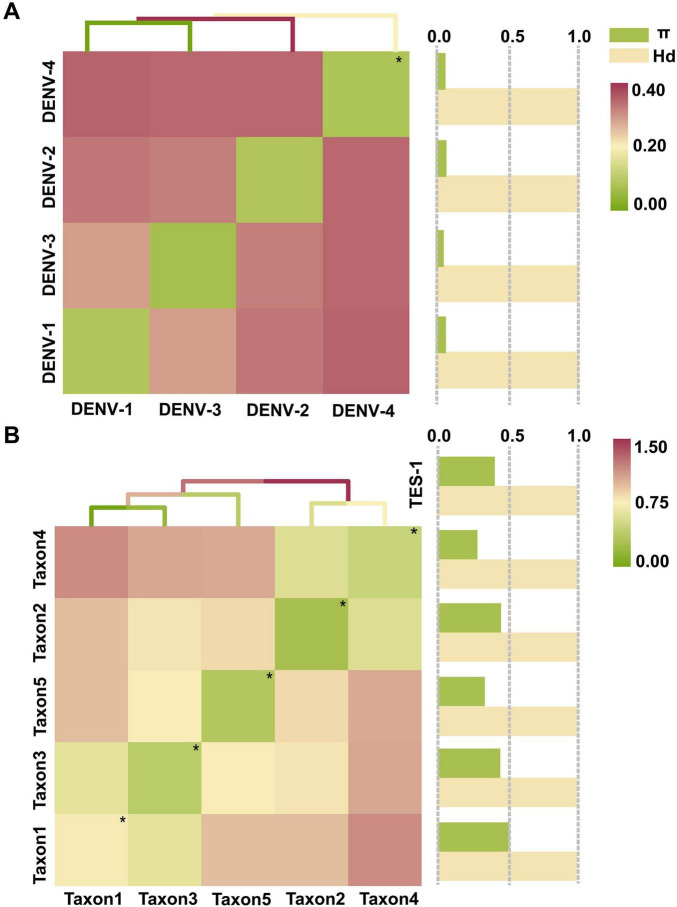
The tests for GEDI matrix and gene flow. **(A)** GEDI matrix and gene flow of TRS. **(B)** GEDI matrix and gene flow of TES-1. The diagonal line of the matrix heat map with “*” and small green spots indicates the average intraserotype GEDI. π and Hd are normalized values (The normalized interval is “[0, 1]”). Hd, Haplotype (gene) diversity; π, Nucleotide diversity.

The maximum interserotype GEDI observed between DENV-1 and DENV-4 was 0.3821, while the overall mean GEDI for the four DENV serotypes was 0.2517 (Figure 5A and Supplementary Table 4, sheet 3). These values were significant because they demonstrated that the genetic divergence between different serotypes (interserotype) was notably higher compared to the divergence observed within the same serotype (intraserotype). Specifically, the maximum interserotype GEDl was 6.0 times greater and the overall mean GEDl was 4.0 times greater than the minimum intraserotype GEDl observed for DENV-2 (0.0632). This high interserotype GEDl indicated greater genetic distance between serotypes, facilitating the use of barcode segments to reliably distinguish between them. Furthermore, GEDI values for TES-1 (0.6840) and taxon3 (0.8073) were 3.8 and 2.4 times greater than that of the overall DENVs group (without differentiation by serotype, 0.1786 and 0.3416, respectively) (Figure 5B and Supplementary Table 4, sheet 3). This indicated that genetic variation among phylogenetically closer groups within the four DENV serotypes was significantly less compared to that of more distantly related groups. Specifically, the GEDI values showed that the four DENV serotypes shared a high level of genetic similarity, whereas the TES-1 and taxon3 groups exhibited higher genetic divergence. These findings suggested that the relatively lower genetic variation within the DENV serotypes made it easier to identify and differentiate them using barcode segments, which was crucial for accurate serotype classification and surveillance.

Gene flow assessments revealed that the haplotype diversity (Hd) among the four DENV serotypes was very high, ranging from 0.9995 to 0.9998 (Figure 5A and Supplementary Table 4, sheet 4), indicating almost complete diversification of haplotypes. Similarly, diversification among species within the *Flaviviridae* family and genera was showed near complete the diversity of haplotype, with Hd values ranging from 0.9910 to 1.0000 (Figure 5B and Supplementary Table 4, sheet 4). Taxa 1, 3, and 4 had nucleotide diversity (π) values of 0.5104, 0.4455, and 0.4503, respectively, which surpassed the overall π value of TES-1 (0.4063). In contrast, taxon2 and taxon5 had lower π values of 0.2838 and 0.3354, respectively, highlighting substantial divergence in genetic variation among *Flaviviridae* species. Specifically, the higher π values in taxa 1, 3, and 4 suggest increased genetic diversity, while the lower values in taxon2 and taxon5 indicate reduced genetic variation, pointing to differing degrees of genetic exchange between these taxa. Furthermore, π values among the four DENV serotypes ranged from 0.0440 to 0.0625, providing evidence of genetic exchange among them.

### Phylogenetic analysis

After thorough examination and alignment with data from the NCBI-Taxonomy database,^18^ it became evident that the *Flavivirus* genus suffered from a convoluted species classification. This genus was populated with many unclassified *Flavivirus* species. These entries compromised the precision and visual clarity of phylogenetic analyses within the *Flaviviridae* family. Considering that the barcode segments designed in our study aimed mainly at identifying DENVs, we opted to temporarily exclude *Flavivirus* species from phylogenetic investigation. The efficacy of barcode segments for differentiating *Flavivirus* species (TES-3-1) would be further deliberated in the subsequent segment “Barcode segments testing”.

**FIGURE 6:**
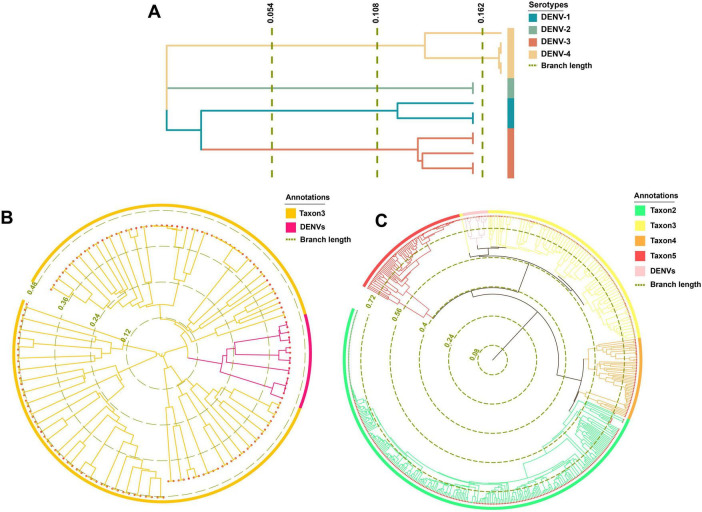
Phylogenetic analysis results. **(A)** The phylogenetic tree of TRS. **(B)** The phylogenetic tree of taxon 3. **(C)** The phylogenetic tree of TES-1.

The maximum likelihood phylogenetic trees offered superior species classification with unambiguous branches (Figures 6A–C). In contrast, the Bayesian tree was suboptimal with fewer counts (Supplementary Figure 1). Phylogenetic results highlighted a pronounced differentiation boundary for DENVs at the serotype level and at the genus or family levels. These findings suggested the presence of stable molecular genetic markers within DENVs, such as specific non-coding regions and conserved structural protein genes, that distinguished between different serotypes and other closely related species, influencing their adaptive evolution over the long term. Phylogenetically, DENV-1 and DENV-3 shared a closer relationship compared to DENV-2 and DENV-4 (Figure 6A). The distant branches and greater divergence from the common ancestor suggested that DENV-2 and DENV-4 had a stronger trend toward species expansion and robust adaptive evolutionary potential. In Figure 6B, the circular phylogenetic tree revealed that the evolutionary distance between DENVs and Taxon3 was relatively small, suggesting that they might share a common ancestor or have undergone similar environmental selective pressures. The red branches representing DENVs were clearly clustered together, indicating minimal genetic divergence among them and suggesting they could be classified as a single group, reflecting the close relationships among the four serotypes. The observed clustering of Taxon3 and DENVs suggested a shared evolutionary history or the retention of similar characteristics within their genomes. This phenomenon implied that these species might have experienced similar selective pressures or horizontal gene transfer events (e.g., genome integration), resulting in comparable genetic traits. Figure 6C depicted the complex evolutionary relationships among different taxa using a circular phylogenetic tree. Several longer branches indicated that these species or taxa had undergone significant evolutionary divergence, likely due to different environmental selective pressures or events such as genome recombination. In contrast, shorter branches represented smaller differences between species, which commonly occurred within the same group, such as the DENV population. The clustering of DENVs in the phylogenetic tree suggested minimal genetic variation among them, while their proximity to other groups (e.g., Taxon3 and Taxon5) might indicate a shared evolutionary background (Postel et al., 2021). As DENVs have evolved to enhance specific virulence factors, such as increased expression of the NS1 protein that aided immune evasion and viral replication, it was crucial to be cautious of increased transmissibility and avoiding cross-infection with different diseases, which could lead to unknown immune evasion scenarios (Zimmerman et al., 2018).

### Visualization of barcode segments

The comprehensive cohort of DENVs contained a total of 9,366 SNP sites. Specifically, DENV-1, DENV-2, DENV-3, and DENV-4 had 6,673, 7,213, 5,985, and 4,943 SNP sites, respectively (Supplementary Table 4, sheet 5). These numbers reflected the genetic variability within each serotype, with DENV-2 showing the highest number of SNP sites, indicating potentially higher evolutionary activity compared to other serotypes. Following the selection framework delineated in the “Barcode segments screening and visualization” section, segments of suboptimal quality were removed. After Blast verification (*P*-value < 0.05), eight SNP-rich barcode segments with high BSW scores were isolated and cataloged (Table 4 and Supplementary Table 5). Based on the criteria of maximal *E*-value, the usage level of barcode segments DENV-1.2 (600) and DENV-4.2 (600) was designated as “Alternative”, while the others were classified as “Optimal”. All barcode segments were in the 3’ untranslated regions according to Blast results on NCBI. Consequently, the identification efficacy of these barcode segments was not compromised by mutations in CDSs or large-scale genomic variations, prolonging their effective duration of use in virus identification studies. The barcode segments of DENV.2 and DENV-2.1, with the highest BSW scores of 5.93 and 5.94 respectively, appeared to be the most suitable for deployment in complex settings, such as metagenomic analyses involving diverse environmental samples or high-throughput sequencing platforms used for large-scale epidemiological studies (Lizarazo et al., 2019).

**TABLE 4:** Basic information of DENV-specific and DENV serotype-specific barcode segments.

Serial numbers	Nucleotide sequence of barcode segments	*P*-value in DnaSP	BSW score	Levels	Location
DENV.1	AAGGACTAGAGGTTAGAGGAGACCCCCC	1.00E−04	5.72	Optimal	3′ untranslated regions
DENV.2	AAACAGCATATTGACGCTGGGAGAGACCAGAGATCCTGCTGTCTC	1.00E−04	5.93	Optimal	3′ untranslated regions
DENV-1.1	AAGAGCTATGCTGCCTGTGAGCCCCGT	1.00E−04	5.73	Optimal	3′ untranslated regions
DENV-1.2	CCGTCTTTCAATATGCT	1.00E−04	5.53	Alternative	3′ untranslated regions
DENV-2.1	CTAGCGGTTAGAGGAGACCCCTCCCT	1.00E−04	5.94	Optimal	3′ untranslated regions
DENV-3.1	TCAATATGCTGAAACGCGTGAGAAACCG	1.00E−04	5.75	Optimal	3′ untranslated regions
DENV-4.1	CCACCTTTCAATATGCTGAAACGCGAGAGAAACCGCGTATC	1.00E−04	5.89	Optimal	3′ untranslated regions
DENV-4.2	GTGCTGCCTGTAGCTCCGCC	1.00E−04	5.60	Alternative	3′ untranslated regions

**FIGURE 7:**
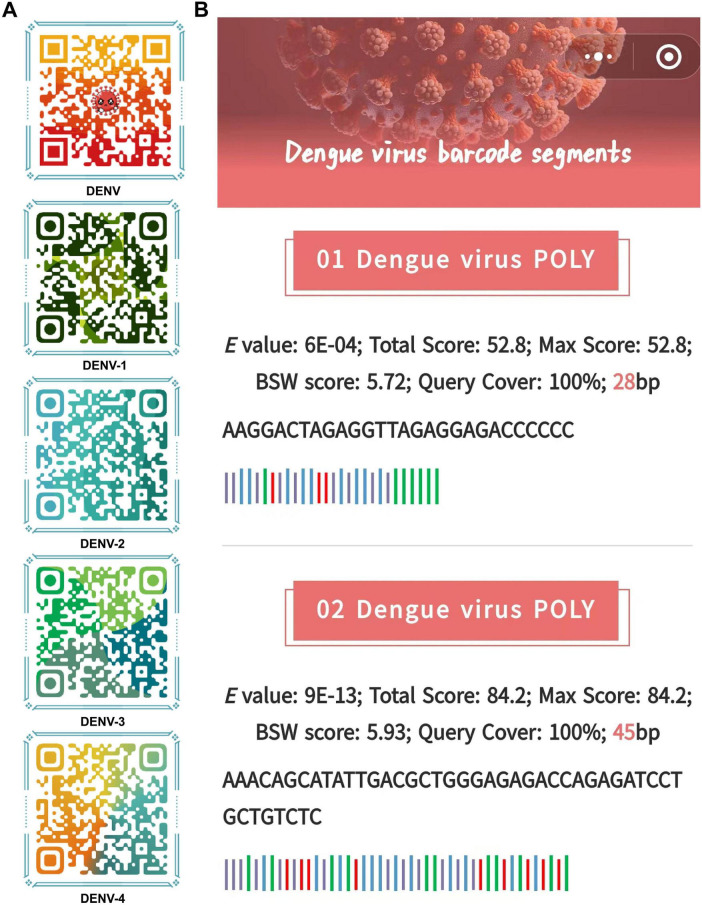
The visual dynamic 1D and 2D codes. **(A)** 2D code format for storing barcode segments. Examples of 1D code format for storing barcode segments. **(B)** People can scan these 2D codes to obtain barcode segments stored in the 1D codes and basic information about barcode segments. The barcode segments use the standard representation of DNA sequences, which is denoted as ACGT.

Ultimately, barcode segments were visualized as a combination of 1D and 2D codes, which together provided comprehensive information about the sequences and allowed efficient data retrieval. The 1D codes, presented in text format, displayed essential information such as sequence length, base composition, and Blast result parameters in a clear and intuitive manner (Figure 7A). The inclusion of 2D codes facilitated rapid information retrieval by allowing users to scan the code using mobile devices or scanners to directly access sequence annotations and Blast analysis results, providing an efficient means of sharing and accessing barcode information (Figure 7B). This dual coding approach enhanced accessibility and made data interpretation faster and more user-friendly, especially in field research and laboratory settings where rapid information exchange was critical.

### Validation of barcode segment primers and experimental workflow

Moreover, primer tests for all barcode segments were conducted to ensure their specificity and accuracy. These tests included *in silico* PCR simulations and BLAST analyses against a comprehensive nucleotide database to confirm that the predicted amplification products matched only the intended target species, without cross-reactivity. Metrics such as primer efficiency (> 90%) and specificity (no off-target amplification with *P*-value < 0.01) were used to confirm their accuracy. Details of these barcode segment primers were available in Supplementary Table 6.

**FIGURE 8:**
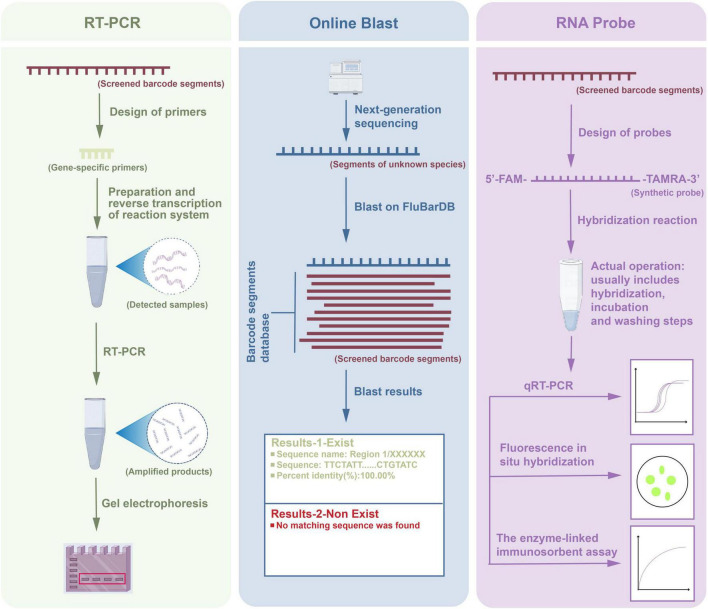
Workflow diagram for RT-PCR, Blast, and RNA probe applications on barcode segments.

In this study, we conducted multiple parallel application experiments on the target barcode segments, as shown in Figure 8. The workflow consisted of three parts: RT-PCR amplification, online Blast analysis, and RNA probe detection, each independently applied for the detection and analysis of barcode segments. First, specific primers were designed for the screened barcode segments, and the reaction system was prepared to reverse transcribe the RNA from the samples into complementary DNA. Subsequently, PCR was performed to amplify the specific gene segments, and the amplified products were verified by gel electrophoresis to ensure successful and specific amplification. In the online Blast analysis, the amplified products were sequenced using next-generation sequencing technology, followed by a Blast search against the DBaaS database. The Blast results indicated that if a matching barcode sequence existed, detailed alignment information was provided, including the sequence name, matched segment, and percentage identity. If no match was found, the result was recorded as “No matching sequence found.” This step helped to confirm whether the target barcode segment was present in the sample. For the RNA probe detection part, we designed and synthesized RNA probes labeled with 5’-FAM and 3’-TAMRA, which hybridized with the target RNA segments in the samples. The hybridization reaction included incubation and washing steps to ensure specificity. Subsequently, three techniques, qRT-PCR, fluorescence *in situ* hybridization, and enzyme-linked immunosorbent assay were employed to analyze the target segments quantitatively and spatially. qRT-PCR was used for precise quantification of the target sequence expression, fluorescence *in situ* hybridization enabled spatial localization of RNA segments in the samples, and enzyme-linked immunosorbent assay further verified the concentration of specific segments in the samples.

### Barcode segments testing

**FIGURE 9:**
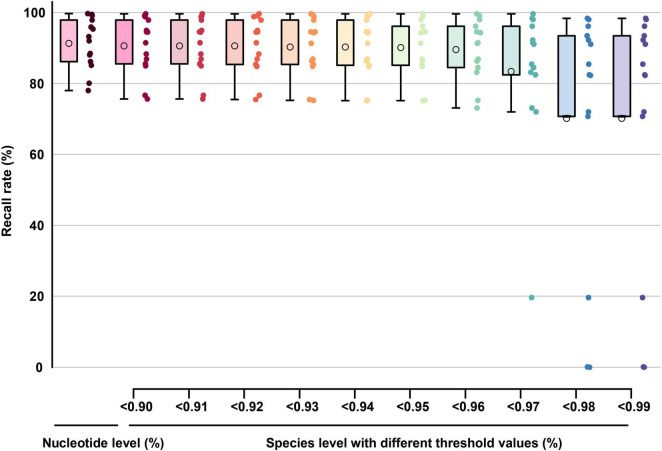
The boxplot graph of the recall rate (TES-2) at nucleotide-level and species-level of barcode segments. The circles inside each box represent the mean values of each data group.

In TES-2, the average nucleotide-level recall rate for all barcode segments reached 91.34% with Barcoding software (Figure 9). This percentage was derived by calculating the average number of correctly recalled nucleotides across multiple sequencing tests, accounting for both high-quality and low-quality samples. Barcode segments DENV-3.1, DENV-4.1, and DENV-4.2 achieved similarly high recall rate (Supplementary Table 2, sheet 2), demonstrating the effectiveness of these barcode segments in identifying new DENV variants or those with lower sequencing quality. When the threshold for recall rate was set above 0.90, the average recall rate for all barcode segments was approximately 90.63% at the species level. This threshold indicated that when the nucleotide composition or sequence similarity between the barcode segment and the sample exceeded 90%, the accuracy of species identification reached 90.63%. This high threshold ensured robust performance even with moderate variability in sequence quality. Furthermore, when the threshold was set above 0.99, the average recall rate at the species level remained above 70.00%, except for DENV.1 (subject: DENV-2) and DENV.2 (subject: DENV-2 and DENV-4). The 70.00% threshold demonstrated that the barcode segments could provide reliable identification even for sequences that are more divergent from the reference, although the recall rate decreases due to increased genetic variability. Even for sequences with poor quality containing gaps (which could interfere with recall rate, Supplementary Table 2, sheet 2), the barcode segments still provided high coverage and a recall rate greater than 70.00%. This indicated that the barcoding system was resilient to sequence imperfections and can maintain a reasonable level of identification accuracy despite sequencing errors or missing data.

**FIGURE 10:**
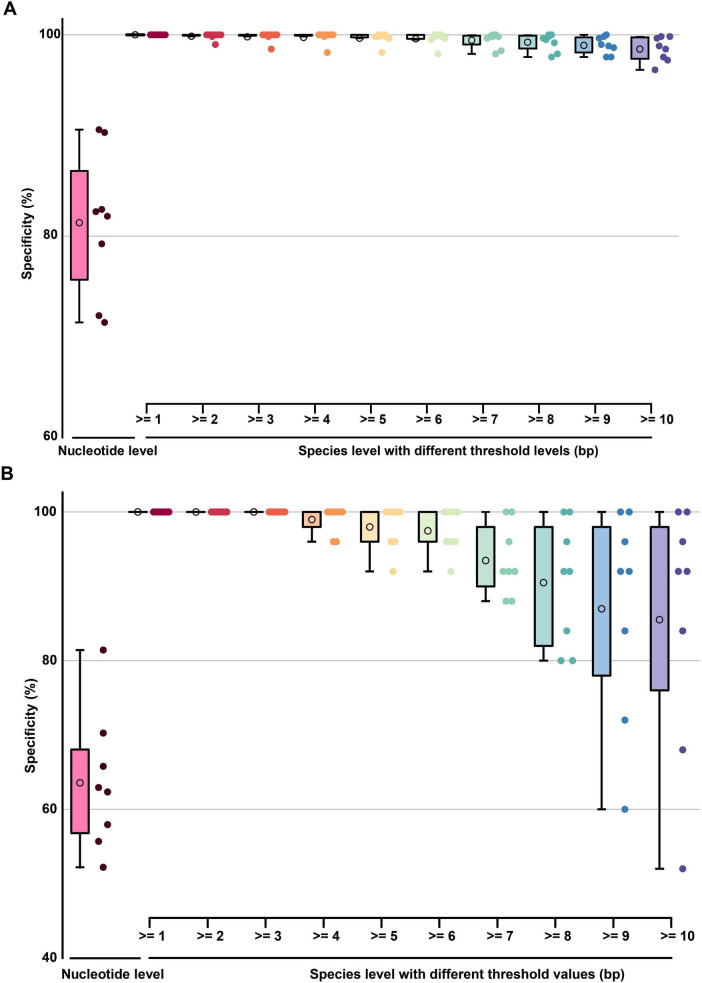
The boxplot graph of the specificity of barcode segments. **(A)** The boxplot graph of the specificity (TES-3-1) at nucleotide-level and species-level of barcode segments. **(B)** The boxplot graph of the specificity (TES-3-2) at nucleotide-level and species-level of barcode segments. The circles inside each box represent the mean values of each data group.

The average nucleotide-level specificity for TES-3-1 across all evaluated barcode segments ranged from 71.43% to 90.59%, with a mean of 81.34% (Figure 10A and Supplementary Table 2, sheet 3), thereby allowing reliable discrimination from non-target species. This indicated that DENVs had an average nucleotide divergence of over 81.34% from closely related strains in these regions, meaning that most of the nucleotide positions differed from those of phylogenetically related species, enabling effective identification. The average specificity of the barcode segments at the species level (base pair resolution) was 99.50%, indicating a high ability to correctly identify species based on unique nucleotide signatures. Notably, even with a threshold set to ≥ 10bp, all barcode segments achieved a remarkable identification accuracy of 98.58% across taxa within the same family. indicating a high ability to correctly identify species based on unique nucleotide signatures. For TES-3-2, nucleotide-level specificity ranged from 52.20% to 81.43%, averaging around 63.58% (Figure 10B and Supplementary Table 2, sheet 4). With a threshold of ≥ 3bp, all barcode segments reached 100% specificity in identifying diverse arboviruses. However, shorter thresholds may not provide sufficient discriminatory power between closely related species. Therefore, increasing the threshold to ≥ 10bp allowed 62.50% of barcode segments to differentiate more than 90% of arboviruses with high specificity. The choice of the 10bp threshold was based on preliminary analyses showing that sequences of this length provide an optimal balance between specificity and sensitivity for distinguishing closely related viruses. This finding highlighted that increasing the sequence length threshold provided better resolution for distinguishing between related species, thereby improving identification reliability. For example, closely related strains such as Zika virus and West Nile virus could be effectively distinguished from DENVs by adjusting the threshold settings. Thus, TES-3-2 results confirmed that species-specific barcode segments for DENVs exhibited nucleotide differences from DENVs and their phylogenetic relatives, and demonstrated that closely related strains could still be effectively distinguished by adjusting the threshold settings.

Given the reliance on NCBI data for selecting the barcode segments and Blast analyses conducted in this study, the TES-4 series (types of strains and their collection details from different databases were provided in Table 5) were primarily used to investigate the generalization capability of the barcode segments in recognizing DENVs or differentiating other strains within new data sources (Supplementary Table 2, sheets 5–9). Results from TES-4-1, TES-4-4, and TES-4-5 demonstrated a 0% false omission rate. The false omission rate was calculated as the proportion of false negatives among all negative predictions, meaning that no target species were incorrectly classified as non-targets, thus confirming the reliability of the barcode segments in identifying SARS-CoV-2 variants, *Coronaviridae* species, *Poxviridae* species, and Monkeypox virus using DENV strain-specific and DENV serotype-specific barcode segments. However, the barcode segment of DENV-1.2 did not yield significant similarity in TES-4-4 at a precision rate of 0%. This lack of precision was primarily caused by high sequence divergence between the DENV-1.2 segment and the sequences present in the TES-4-4 dataset, indicating that this barcode segment may have limited utility in identifying distantly related strains. Furthermore, except for that of DENV.1 (a minimum precision rate of 95.65% and a minimum coverage of 82.00% in TES-4-6), the precision rate and minimum coverage for other barcode segments both reached 100% in TES-4-2, TES-4-3, and TES-4-6. The minimum precision rate of 95.65% for DENV.1 indicated that although this barcode segment showed slightly reduced specificity, it still maintained a high level of accuracy in correctly identifying target species, suggesting robustness in distinguishing between closely related strains despite using advanced algorithms. minor variability.

**TABLE 5:** Generalization testing results.

TESs	Virus types	Time span (collection date)	False omission rate	Precision rate
TES-4-1 (GISAID’s EpiCoV)	All SARS-CoV-2 variants	Up to the 20th of July, 2024	All 0%	–
TES-4-2 (GISAID’s EpiArbo)	Four DENV serotypes	Up to the 20th of July, 2024	–	All 100%
TES-4-3 (BV-BRC)	Reference and representative genomes (virus)	Up to the 20th of July, 2024	–	All 100%
TES-4-4 (NGDC)	*Coronaviridae* famliy, *Poxviridae* family, Monkeypox virus genomes, NCBI RefSeq viruses representation genomes	Up to the 8th of January, 2024	All 0% except DENV-1.2 (no results)	–
TES-4-5 (NCBI-influenza)	Influenza A, B and C viruses	Up to the 20th of July, 2024	All 0%	–
TES-4-6 (RVDB)	Nucleotide database	Up to the 22th of November, 2023	–	All 100% except DENV.1 (min precision rate is 95.65% and min coverage is 82.00%)

## Discussion

Over the past two decades, molecular genetic markers such as barcode segments, short tandem repeats, microsatellites, and metabarcoding has proven useful in taxonomic classification (Yu, 2023), species identification, and diversity assessments across various organisms, including animals (Tanudisastro et al., 2024; Lai et al., 2022), plants (Li et al., 2021), and microbes (You et al., 2024). Moreover, these markers have significantly contributed to our understanding of phenotypic variations by enabling the identification of specific genetic variants associated with observable traits, such as flower pigmentation in *Orchidaceae* and pathogenicity markers in *Coronaviridae* (Tanudisastro et al., 2024; You et al., 2024). Additionally, they have been instrumental in predicting disease risks, such as the susceptibility of certain *Theaceae* species to fungal pathogens, demonstrating substantial diagnostic and scientific potential through the application of variant calling techniques. In studying barcode segments on species in *Theaceae*, *Orchidaceae* and *Coronaviridae* families, researchers have combined molecular biology and bioinformatics approaches and algorithms to screen for species-specific barcode segments using advanced algorithms. The stability and reliability of species recognition by barcode segments has been corroborated by GEDI matrices and phylogenetic tree results, which consistently showed clear differentiation between species.

With the increasing application of short-read exomes and whole-genome sequencing in clinical contexts, screening species-specific barcode segments within whole-genome datasets was more advantageous than conducting multiple genetic tests (Bartha and Gyõrffy, 2019). This study was among the first to utilize whole-genome data from all four DENV serotypes to screen barcode segments, providing a more comprehensive approach compared to previous studies that focused on partial genomic regions or individual genes (Li et al., 2021; Jiang et al., 2022). A total of eight barcode segments were identified, covering both intra- and interserotype specificity, including two segments for DENV, two for DENV-1, one for DENV-2, one for DENV-3, and two for DENV-4. GEDI, phylogenetic analysis, and TES results indicated barcode segments could specifically identify DENVs and distinguish between different serotypes with high precision. Furthermore, the barcode segments were visualized using 1D and 2D codes, offering efficient readability, ease of understanding, and enhanced recognition. This dual-coding system supported greater portability and accessibility, facilitating the adoption of barcoding technology, especially in contexts where users may have limited experience with genomic data interpretation (Kress et al., 2015). The online barcode sharing platform integrated barcode segment-related datasets and made them publicly available through a dedicated web portal, providing downloadable resources and comprehensive documentation (Harris et al., 2019). This public availability addresses the existing gap in DENV barcode segment databases, thereby enhancing global information sharing and fostering collaboration among researchers, industry, and healthcare practitioners. The accessible platform inspires new research projects and promotes technological innovations by providing valuable tools for community-driven genomic analysis.

Based on previous research (You et al., 2024; Li et al., 2021; Jiang et al., 2022), we proposed three new requirements for constructing TRS: ease of building, batch processability, and high versatility. These requirements were determined based on limitations observed in existing reference sets, where difficulties in building comprehensive datasets, lack of scalability for batch processing, and limited adaptability to new data sources posed significant challenges. Our aim was to overcome these limitations and make the TRS more suitable for real-world applications. Firstly, sequences were collected based on search criteria, including reference sequences from the RefSeq database and published sequences, ensuring high-quality and representative datasets. Sequencing accuracy was ensured by using reference sequences that met minimum quality scores (e.g., Q30 or higher), which indicates a 99.9% confidence level for correct base calls. Additionally, NCBI annotations of the relative positions of CDSs were used, facilitating horizontal comparison of CDSs within the four DENV serotypes. The TRS also included many “prominent” strains with extensive global impact, such as the Zika virus and Hepatitis C virus, enhancing the representativeness of barcode segments by including strains known for their global epidemiological significance (Weger-Lucarelli et al., 2018). Secondly, the TRS’s internal structure was designed in a modular manner, allowing for batch modifications of one or multiple modules with strong scalability. This modular design was achieved by organizing the TRS into distinct functional units, such as genomic regions or taxonomic categories, which could be independently updated or replaced. This structure enabled researchers to easily update the TRS in batches as new data becomes available or as research needs evolve (Newman, 2006). Thirdly, to address compatibility issues with RNA viral nucleotide sequences in some databases or software, we developed three schemes to enhance TRS versatility: 1) Format adjustments: partially adjusting the format of output files (e.g., converting base U to T) according to NCBI standards (You et al., 2024), improving compatibility with different bioinformatics tools; 2) Genetic noise reduction: removing all degenerate bases within TRS to reduce genetic noise and minimize alignment errors, thereby improving the quality of comparative analyses; and 3) Multi-level classification: establishing multiple classification levels or categories (termed multi-level taxa) for key species under study, especially those with multiple serotypes, subtypes, sublineages, or subspecies. This structure enabled more detailed and nuanced analysis of viral diversity, particularly for viruses with complex taxonomies (You et al., 2024).

The SNP and population genetic studies of TRS and TES-1 provided vital insights into DENV variability, specifically highlighting high levels of genetic diversity within serotypes, evidence of intra-serotype gene flow, and the presence of unique SNP markers that distinguish between different DENV serotypes. These findings aided in understanding their evolutionary dynamics and explaining persistent dengue outbreaks. Frequent gene flow enhanced biodiversity within virus populations, suggesting that the transmission of *Flaviviridae* species was largely unrestricted by time and space (Samuk and Noor, 2022). DENVs have evolved into a complexly structured group, allowing exploration of their evolutionary relationships with other *Flaviviridae* species through SNP analysis (Bamford et al., 2022). Gene flow results showed potential genetic material transmission between *Orthoflavivirus* and *Pestivirus* genera. *Orthoflavivirus* included human pathogens like DENVs and yellow fever virus, while *Pestivirus* was associated with animal diseases like bovine viral diarrhea and classical swine fever (Postel et al., 2021). Recombination and gene splicing played critical roles in shaping viral genomes by creating new genetic combinations that could lead to increased adaptability, altered virulence, or expanded host range. For instance, recombination events between *Orthoflavivirus* and *Pestivirus* have been reported to result in increased virulence and the development of new phenotypes that can escape host immunity (Weber et al., 2015). The involvement of intermediate hosts in mediating genetic exchanges between genera could facilitate the emergence of more adaptable strains, as seen in other flaviviruses where recombination has led to variants with enhanced transmission efficiency and virulence (Chu et al., 2022).

Due to the high antigenic variability and widespread infective capability of *Flaviviridae* species, phylogenetic analyses have facilitated a deeper understanding of their transmission pathways, host adaptation mechanisms, and resistance traits (Bell et al., 2019). For example, phylogenetic analysis has elucidated the spread of DENV serotypes across different geographic regions and identified genetic markers associated with resistance to vector control measures. Phylogenetic outcomes suggested minimal impact on species classification due to elongated branches resulting from sequences with low coverage, high error rates, or incomplete genome data. Despite these inconsistencies, the overall tree topology remained robust, indicating that the dataset still provided a reliable representation of evolutionary relationships. Like *Betacoronavirus* and HCoVs (You et al., 2024), DENVs harbored abundant SNP sites that function like internal nodes of a phylogenetic tree, effectively aiding in identifying closely related species (e.g., *Hepacivirus* species). The high genetic variability observed in DENVs was driven by the high mutation rates of their RNA genomes and genetic rearrangements (e.g., gene flow). External selective pressures, such as host immune responses and vector-host interactions, played a more significant role in shaping DENV evolution compared to neutral evolutionary forces (Frost et al., 2018). This was supported by the presence of specific genetic adaptations that facilitated immune escape and enhanced vector transmission, indicating strong selection rather than random genetic drift. For these reasons, this study emphasized exploring genetic diversity in DENVs through gene flow tests rather than focusing on neutral evolution models. Gene flow results revealed substantial genetic material exchange among different DENV serotypes, which facilitated genetic recombination and increased diversity within populations. This exchange was particularly significant in mixed infection areas, where multiple DENV serotypes co-circulate, leading to the emergence of genetically diverse viral strains (You et al., 2024). By examining gene flow, we could better understand the dynamics of DENV spread, cross-species transmission, and the potential for emergence of new, more virulent strains. The focus on gene flow provided insights into temporal and geographical trends of DENV evolution, emphasizing the importance of regional control measures to prevent the spread of diverse and potentially more dangerous strains (Welch et al., 2008; Samuk and Noor, 2022).

Considering the genetic differentiation among the four DENV serotypes, this study employed various algorithms to ensure the robustness of barcode segments. By treating these serotypes as independent or consolidated units, we minimized the loss of conservative nucleotide sequences due to alignment gaps or missing data, optimizing the process of selecting barcode segments. For the first time, we utilized logarithmic weighting results from NCBI and DnaSP v6 to derive BSW values and evaluated the accuracy and stability of barcode segment identification (You et al., 2024). The barcode segments highly overlapped with conserved regions (Zhao et al., 2003), indicating their reliability as molecular markers (Blois et al., 2022). Compared to barcoding technology for identifying SARS-CoV-2 (average specificity: 29.38%) or strains relating to SARS-CoV and SARSr-CoV-2 strains (You et al., 2024; Guan et al., 2020), the recall rate and specificity for DENV barcode segments in this study were higher. Additionally, the process of selecting barcode segments in this study demonstrated improved data processing speed and lower computational resource consumption. Specifically, the optimized workflow achieved a 25% reduction in computational time compared to previous barcoding methods (You et al., 2024). This efficiency in data processing, combined with maintaining high identification accuracy, made the approach particularly suitable for resource-limited laboratories and critical for rapid response to public health incidents where both speed and accuracy were essential.

The TESs encompassed sequences of numerous strains from databases, including *Flaviviridae* species as well as pathogens of domestic and international concern such as monkeypox virus, SARS-CoV-2, African swine fever virus, equine encephalitis virus, and Getah virus (Gaudreault et al., 2020). TESs also included common arboviruses that pose specific threats to human and animal health, including their ability to cause severe economic losses in livestock and public health impacts in humans. For example, African swine fever led to high mortality rates in pigs, affecting agriculture, while equine encephalitis could cause severe neurological disease in horses and humans. These harmful factors were not limited to their geographic distribution (tropical and subtropical regions) and transmission vectors (e.g., blood-feeding arthropods), but also extended to positioning arboviruses as a species group spanning multiple families such as *Asfarviridae* (Outammassine et al., 2022). Phylogenetic analyses indicated that DENVs shared closer relationships with certain arboviruses, such as West Nile virus and Japanese encephalitis virus, than with most other members of the *Flaviviridae* family. These findings suggested that evolutionary pressures and transmission dynamics may have led to convergent adaptations among these viruses. The inclusion of arboviruses in the TES for verifying barcode segment specificity was uncommon in previous studies, thus enhancing the robustness of our approach (Batovska et al., 2018). Additionally, the strains included in TESs were extensive, spanning from the 1990s to 2024, and featured viruses involved in several major epidemic outbreaks including SARS (2002 - 2003), H1N1 influenza (2009 - 2010), Zika virus outbreak (2015 - 2016), and COVID-19 (late 2019 - ongoing as of 2023). This broad temporal range allowed us to assess the stability of the barcode segments across diverse temporal and geographic contexts, providing comprehensive verification of their reliability. The ongoing status of the COVID-19 pandemic was also captured, allowing for continuous monitoring and validation of barcode specificity. The extensive range of strains further confirmed that potential recombination phenomena had minor impact on the stability and reliability of the selected barcode segments (Yu, 2023; Guan et al., 2020). This was evidenced by the high consistency of recall rates and specificity values across diverse datasets, with the recall rate remaining above 90% for most segments, and no significant deviation observed in phylogenetic clustering despite the inclusion of strains with known recombination events. These results indicate that recombination did not disrupt the genetic signatures captured by the barcode segments. This consistency supported the robustness of the barcode segments in distinguishing DENV serotypes even in the presence of recombination.

Building on the visualization methods of Li et al. (2021), Gogoi et al. (2020), this study modified the color and shape features in 1D codes to enhance the visual representation of the base and BPC content of barcode segments (You et al., 2024). The dynamic 2D code technology allowed real-time modifications of directed links or stored information through the backend. Additionally, it enabled tracking data such as scan counts, times, and locations, providing an interactive experience with barcoding technology. The interface following 2D code scanning was enhanced to improve user perception and information literacy. The news and literature section of an online DBaaS, modeled after http://virusbarcodedatabase.top/ (You et al., 2024), offered valuable insights into disease traits, stimulated new research directions, enhanced public health consciousness, promoted community-level preventive measures, and reduced disease transmission risk.

DENV barcode segments provided a simple, effective means for rapid identification of viruses in samples. This was particularly beneficial in resource-limited settings or during outbreak scenarios where timely detection was crucial for effective intervention. These barcode segments could serve as templates for designing highly specific detection primers, thereby improving the sensitivity and specificity of diagnostic methods, such as RT-PCR, for detecting DENV serotypes. These barcode segments could also be used to filter high-throughput sequencing data, facilitating the identification of DENV sequences in complex environmental samples. This application reduced the complexity and enhanced the efficiency of analyzing large-scale sequencing data, aiding in effective surveillance and monitoring of DENV circulation. Besides, while this study demonstrated the potential of DENV barcode segments in identification through computational analysis, these results were based solely on *in silico* methods at the data level. The lack of wet lab validation might limit our comprehensive understanding of the performance of these barcode segments in practical applications. Therefore, future research will focus on testing these barcode segments using standard samples at the wet lab level. This will help evaluate their sensitivity, specificity, and operability in real-world settings, thereby verifying their practical value in dengue surveillance and diagnosis.

## Conclusion

This investigation developed species-specific barcode segments for efficiently identifying DENVs and their four serotypes within complex matrices, evaluating the accuracy and robustness of these segments. Our study marked the inaugural use of extensive data analysis to construct a sequence database (TRS), leading to the selection of eight specific barcode segments for both intraserotype and interserotype variations of DENVs. These segments were visualized through a composite code (1D and 2D codes) and disseminated publicly. Custom-developed software facilitated numerous bioinformatics experiments, markedly improving data analysis efficiency. Access to insights into DENV genetic diversity and barcode segments via an online platform significantly enriched public understanding of DENVs. Unlike existing barcoding methodologies, our approach underwent rigorous iterative testing and refinement across sequence aggregation, TRS and TES development, genetic diversity examination, and barcode segment selection. This rigorous process yielded a standardized, exhaustive, and replicable framework for barcode segment formulation, providing invaluable technical insights for healthcare professionals. Looking ahead, we aimed to amass a larger corpus of viral data and establish a more comprehensive viral barcode repository, bolstering researchers’ capabilities to devise more rapid and effective therapeutic strategies against a spectrum of viruses.

## Data Availability

The original contributions presented in this study are included in this article/Supplementary material, further inquiries can be directed to the corresponding authors.

## Footnotes

1
http://virusbarcodedatabase.top/denguevirus/

2
https://www.who.int/emergencies/disease-outbreak-news/item/2023-DON498

3
https://www.ncbi.nlm.nih.gov/

4
https://www.ncbi.nlm.nih.gov/datasets/taxonomy/tree/

5
https://ictv.global/taxonomy

6
https://mafft.cbrc.jp/alignment/server/

7
https://www.ncbi.nlm.nih.gov/datasets/genome/?taxon=3052464

8
https://itol.embl.de/

9
https://blast.ncbi.nlm.nih.gov/Blast.cgi

10
https://www.qr-code-generator.com/

11
https://www.ncbi.nlm.nih.gov/tools/primer-blast/index.cgi

12
https://gisaid.org/

13
https://www.bv-brc.org/app/Homology

14
https://ngdc.cncb.ac.cn/blast/blastn

15
https://www.ncbi.nlm.nih.gov/genomes/FLU/Database/nph-select.cgi

16
https://rvdb.dbi.udel.edu/blast

17
https://github.com/Tetingp/Barcoding1/releases/tag/Barcoding1

18
https://www.ncbi.nlm.nih.gov/Taxonomy/Browser/wwwtax.cgi?mode=Undef&id=11050&lvl=3&lin=f&keep=1&srchmode=1&unlock

## Supplementary material

Supplementary Figure 1 The Bayesian phylogenetic tree of TRS.

Supplementary Table 1 Detailed information (including accession numbers, version numbers, length, collection date, update date and search details) of sequences in TRS and phylogenetic analysis (Figure 6A).

Supplementary Table 2 Detailed TESs’ results for barcode segments.

Supplementary Table 3 The configuration of network environment architecture.

Supplementary Table 4 The nucleotide polymorphism and genetic features of TRS and TES-1.

Supplementary Table 5 The basic information and Blast results of barcode segments.

Supplementary Table 6 The primer information of barcode segments.

## References

[B1] Afroz S. Giddaluru J. Abbas M. M. Khan N. (2016). Transcriptome meta-analysis reveals a dysregulation in extra cellular matrix and cell junction associated gene signatures during Dengue virus infection. *Sci. Rep.* 6:33752. 10.1038/srep33752 27651116 PMC5030657

[B2] Agosto-Arroyo E. Coshatt G. M. Winokur T. S. Harada S. Park S. L. (2017). Alchemy: a web 2.0 real-time quality assurance platform for human immunodeficiency virus, Hepatitis C virus, and bk virus quantitation assays. *J. Pathol. Inform.* 8:18. 10.4103/jpi.jpi_69_16 28480121 PMC5404607

[B3] Altschul S. F. Madden T. L. Schäffer A. A. Zhang J. Zhang Z. Miller W. (1997). Gapped BLAST and PSI-BLAST: a new generation of protein database search programs. *Nucleic Acids Res.* 25 3389–3402. 10.1093/nar/25.17.3389 9254694 PMC146917

[B4] Álvarez-Díaz D. A. Valencia-Álvarez E. Rivera J. A. Rengifo A. C. Usme-Ciro J. A. Peláez-Carvajal D. (2021). An updated RT-qPCR assay for the simultaneous detection and quantification of chikungunya, dengue and zika viruses. *Infect. Genet. Evol.* 93:104967. 10.1016/j.meegid.2021.104967 34116240

[B5] Bamford C. G. G. de Souza W. M. Parry R. Gifford R. J. (2022). Comparative analysis of genome-encoded viral sequences reveals the evolutionary history of flavivirids (family *Flaviviridae*). *Virus Evol.* 8:veac085. 10.1093/ve/veac085 36533146 PMC9752770

[B6] Bao Y. Bolotov P. Dernovoy D. Kiryutin B. Zaslavsky L. Tatusova T. (2008). The influenza virus resource at the National Center for Biotechnology Information. *J. Virol.* 82 596–601. 10.1128/jvi.02005-07 17942553 PMC2224563

[B7] Bartha Á Gyõrffy B. (2019). Comprehensive outline of whole exome sequencing data analysis tools available in clinical oncology. *Cancers (Basel)* 11:1725. 10.3390/cancers11111725 31690036 PMC6895801

[B8] Batovska J. Lynch S. E. Cogan N. O. I. Brown K. Darbro J. M. Kho E. A. (2018). Effective mosquito and arbovirus surveillance using metabarcoding. *Mol. Ecol. Resour.* 18 32–40. 10.1111/1755-0998.12682 28417591 PMC5811807

[B9] Bell S. M. Katzelnick L. Bedford T. (2019). Dengue genetic divergence generates within-serotype antigenic variation, but serotypes dominate evolutionary dynamics. *Elife* 8:e42496. 10.7554/eLife.42496 31385805 PMC6731059

[B10] Blois S. Goetz B. M. Bull J. J. Sullivan C. S. (2022). Interpreting and de-noising genetically engineered barcodes in a DNA virus. *PLoS Comput. Biol.* 18:e1010131. 10.1371/journal.pcbi.1010131 36413582 PMC9725130

[B11] Bobay L. M. Ochman H. (2017). Impact of recombination on the base composition of bacteria and archaea. *Mol. Biol. Evol.* 34 2627–2636. 10.1093/molbev/msx189 28957503 PMC5850839

[B12] Boolchandani M. D’Souza A. W. Dantas G. (2019). Sequencing-based methods and resources to study antimicrobial resistance. *Nat. Rev. Genet.* 20 356–370. 10.1038/s41576-019-0108-4 30886350 PMC6525649

[B13] Brillet K. Janczuk-Richter M. Poon A. Laukart-Bradley J. Ennifar E. Lebars I. (2024). Characterization of SLA RNA promoter from dengue virus and its interaction with the viral non-structural NS5 protein. *Biochimie* 222 87–100. 10.1016/j.biochi.2024.02.005 38408720

[B14] Cattarino L. Rodriguez-Barraquer I. Imai N. Cummings D. A. T. Ferguson N. M. (2020). Mapping global variation in dengue transmission intensity. *Sci. Transl. Med.* 12:eaax4144. 10.1126/scitranslmed.aax4144 31996463

[B15] Chu H. M. Liu J. X. Zhang K. Zheng C. H. Wang J. Kong X. Z. (2022). A binary biclustering algorithm based on the adjacency difference matrix for gene expression data analysis. *BMC Bioinform.* 23:381. 10.1186/s12859-022-04842-4 36123637 PMC9484244

[B16] CNCB-NGDC Members and Partners. (2023). Database resources of the national genomics data center, China national center for bioinformation in 2023. *Nucleic Acids Res.* 51 D18–D28. 10.1093/nar/gkac1073 36420893 PMC9825504

[B17] Federhen S. (2012). The NCBI Taxonomy database. *Nucleic Acids Res.* 40 D136–D143. 10.1093/nar/gkr1178 22139910 PMC3245000

[B18] Focosi D. Maggi F. (2022). Recombination in coronaviruses, with a focus on SARS-CoV-2. *Viruses* 14:1239. 10.3390/v14061239 35746710 PMC9228924

[B19] Friedel M. van Eede M. C. Pipitone J. Chakravarty M. M. Lerch J. P. (2014). Pydpiper: a flexible toolkit for constructing novel registration pipelines. *Front. Neuroinform.* 8:67. 10.3389/fninf.2014.00067 25126069 PMC4115634

[B20] Frost S. D. W. Magalis B. R. Kosakovsky Pond S. L. (2018). Neutral theory and rapidly evolving viral pathogens. *Mol. Biol. Evol.* 35 1348–1354. 10.1093/molbev/msy088 29688481 PMC6279309

[B21] Galula J. U. Salem G. M. Destura R. V. Remenyi R. Chao D. Y. (2021). Comparable accuracies of nonstructural protein 1- and envelope protein-based enzyme-linked immunosorbent assays in detecting anti-dengue immunoglobulin G antibodies. *Diagnostics (Basel)* 11:741. 10.3390/diagnostics11050741 33919324 PMC8143319

[B22] Gaudreault N. N. Madden D. W. Wilson W. C. Trujillo J. D. Richt J. A. (2020). African swine fever virus: an emerging DNA arbovirus. *Front. Vet. Sci.* 7:215. 10.3389/fvets.2020.00215 32478103 PMC7237725

[B23] Gogoi B. Wann S. B. Saikia S. P. (2020). DNA barcodes for delineating *Clerodendrum* species of North East India. *Sci. Rep.* 10:13490. 10.1038/s41598-020-70405-3 32778674 PMC7417596

[B24] Goodacre N. Aljanahi A. Nandakumar S. Mikailov M. Khan A. S. (2018). A Reference Viral Database (RVDB) to enhance bioinformatics analysis of high-throughput sequencing for novel virus detection. *mSphere* 3 e00069–18. 10.1128/mSphereDirect.00069-18 29564396 PMC5853486

[B25] Guan Q. Sadykov M. Mfarrej S. Hala S. Naeem R. Nugmanova R. (2020). A genetic barcode of SARS-CoV-2 for monitoring global distribution of different clades during the COVID-19 pandemic. *Int. J. Infect. Dis.* 100 216–223. 10.1016/j.ijid.2020.08.052 32841689 PMC7443060

[B26] Harris P. A. Taylor R. Minor B. L. Elliott V. Fernandez M. O’Neal L. (2019). The REDCap consortium: building an international community of software platform partners. *J. Biomed. Inform.* 95:103208. 10.1016/j.jbi.2019.103208 31078660 PMC7254481

[B27] Jiang S. Chen F. Qin P. Xie H. Peng G. Li Y. (2022). The specific DNA barcodes based on chloroplast genes for species identification of *Theaceae* plants. *Physiol. Mol. Biol. Plants* 28 837–848. 10.1007/s12298-022-01175-7 35592487 PMC9110604

[B28] Johnson B. W. Russell B. J. Lanciotti R. S. (2005). Serotype-specific detection of dengue viruses in a fourplex real-time reverse transcriptase PCR assay. *J. Clin. Microbiol.* 43 4977–4983. 10.1128/jcm.43.10.4977-4983.2005 16207951 PMC1248506

[B29] Kok B. H. Lim H. T. Lim C. P. Lai N. S. Leow C. Y. Leow C. H. (2023). Dengue virus infection - a review of pathogenesis, vaccines, diagnosis and therapy. *Virus Res.* 324:199018. 10.1016/j.virusres.2022.199018 36493993 PMC10194131

[B30] Kress W. J. García-Robledo C. Uriarte M. Erickson D. L. (2015). DNA barcodes for ecology, evolution, and conservation. *Trends Ecol. Evol.* 30 25–35. 10.1016/j.tree.2014.10.008 25468359

[B31] Kumari R. Sharma S. D. Kumar A. Ende Z. Mishina M. Wang Y. (2023). Antiviral approaches against influenza virus. *Clin. Microbiol. Rev.* 36:e0004022. 10.1128/cmr.00040-22 36645300 PMC10035319

[B32] Lai F. Y. Lin Y. C. Ding S. T. Chang C. S. Chao W. L. Wang P. H. (2022). Development of novel microsatellite markers to analyze the genetic structure of dog populations in Taiwan. *Anim. Biosci.* 35 1314–1326. 10.5713/ab.21.0519 35240021 PMC9449399

[B33] Lam T. T. Jia N. Zhang Y. W. Shum M. H. Jiang J. F. Zhu H. C. (2020). Identifying SARS-CoV-2-related coronaviruses in Malayan pangolins. *Nature* 583 282–285. 10.1038/s41586-020-2169-0 32218527

[B34] Letunic I. Bork P. (2024). Interactive Tree of Life (iTOL) v6: recent updates to the phylogenetic tree display and annotation tool. *Nucleic Acids Res*. 52 W78–W82. 10.1093/nar/gkae268 38613393 PMC11223838

[B35] Li H. Xiao W. Tong T. Li Y. Zhang M. Lin X. (2021). The specific DNA barcodes based on chloroplast genes for species identification of *Orchidaceae* plants. *Sci. Rep.* 11:1424. 10.1038/s41598-021-81087-w 33446865 PMC7809279

[B36] Lizarazo E. Couto N. Vincenti-Gonzalez M. Raangs E. C. Velasco Z. Bethencourt S. (2019). Applied shotgun metagenomics approach for the genetic characterization of dengue viruses. *J. Biotechnol.* 306S:100009. 10.1016/j.btecx.2019.100009 34112375

[B37] Marceau C. D. Puschnik A. S. Majzoub K. Ooi Y. S. Brewer S. M. Fuchs G. (2016). Genetic dissection of *Flaviviridae* host factors through genome-scale CRISPR screens. *Nature* 535 159–163. 10.1038/nature18631 27383987 PMC4964798

[B38] Massart S. Chiumenti M. De Jonghe K. Glover R. Haegeman A. Koloniuk I. (2019). Virus detection by high-throughput sequencing of small RNAs: large-scale performance testing of sequence analysis strategies. *Phytopathology* 109 488–497. 10.1094/phyto-02-18-0067-r 30070618

[B39] Newman M. E. (2006). Modularity and community structure in networks. *Proc. Natl. Acad. Sci. U.S.A.* 103 8577–8582. 10.1073/pnas.0601602103 16723398 PMC1482622

[B40] Oh S. Cha J. Ji M. Kang H. Kim S. Heo E. (2015). Architecture design of healthcare software-as-a-service platform for cloud-based clinical decision support service. *Healthc. Inform. Res.* 21 102–110. 10.4258/hir.2015.21.2.102 25995962 PMC4434058

[B41] Olson R. D. Assaf R. Brettin T. Conrad N. Cucinell C. Davis J. J. (2023). Introducing the Bacterial and Viral Bioinformatics Resource Center (BV-BRC): a resource combining PATRIC, IRD and ViPR. *Nucleic Acids Res.* 51 D678–D689. 10.1093/nar/gkac1003 36350631 PMC9825582

[B42] Outammassine A. Zouhair S. Loqman S. (2022). Global potential distribution of three underappreciated arboviruses vectors (*Aedes japonicus*, Aedes vexans and *Aedes vittatus*) under current and future climate conditions. *Transbound. Emerg. Dis.* 69 e1160–e1171. 10.1111/tbed.14404 34821477

[B43] Page A. J. Taylor B. Delaney A. J. Soares J. Seemann T. Keane J. A. (2016). SNP-sites: rapid efficient extraction of SNPs from multi-FASTA alignments. *Microb. Genom.* 2:e000056. 10.1099/mgen.0.000056 28348851 PMC5320690

[B44] Paz-Bailey G. Adams L. E. Deen J. Anderson K. B. Katzelnick L. C. (2024). Dengue. *Lancet* 403 667–682. 10.1016/s0140-6736(23)02576-x 38280388 PMC12372472

[B45] Pomerantz A. Peñafiel N. Arteaga A. Bustamante L. Pichardo F. Coloma L. A. (2018). Real-time DNA barcoding in a rainforest using nanopore sequencing: opportunities for rapid biodiversity assessments and local capacity building. *Gigascience* 7:giy033. 10.1093/gigascience/giy033 29617771 PMC5905381

[B46] Porse C. C. Kramer V. Yoshimizu M. H. Metzger M. Hu R. Padgett K. (2015). Public health response to *Aedes aegypti* and Ae. albopictus mosquitoes invading California, USA. *Emerg. Infect. Dis.* 21 1827–1829. 10.3201/3210.150494 26401891 PMC4593441

[B47] Postel A. Smith D. B. Becher P. (2021). Proposed update to the taxonomy of *Pestiviruses*: eight additional species within the genus *Pestivirus*, family *Flaviviridae*. *Viruses* 13:1542. 10.3390/v13081542 34452407 PMC8402895

[B48] Pruitt K. D. Tatusova T. Maglott D. R. (2005). NCBI Reference Sequence (RefSeq): a curated non-redundant sequence database of genomes, transcripts and proteins. *Nucleic Acids Res.* 33 D501–D504. 10.1093/nar/gki025 15608248 PMC539979

[B49] Rather I. A. Parray H. A. Lone J. B. Paek W. K. Lim J. Bajpai V. K. (2017). Prevention and control strategies to counter dengue virus infection. *Front. Cell. Infect. Microbiol.* 7:336. 10.3389/fcimb.2017.00336 28791258 PMC5524668

[B50] Riaz T. Shehzad W. Viari A. Pompanon F. Taberlet P. Coissac E. (2011). ecoPrimers: inference of new DNA barcode markers from whole genome sequence analysis. *Nucleic Acids Res.* 39:e145. 10.1093/nar/gkr732 21930509 PMC3241669

[B51] Rijnink W. F. Stadlbauer D. Puente-Massaguer E. Okba N. M. A. Kirkpatrick Roubidoux E. Strohmeier S. (2023). Characterization of non-neutralizing human monoclonal antibodies that target the M1 and NP of influenza A viruses. *J. Virol.* 97:e0164622. 10.1128/jvi.01646-22 37916834 PMC10688359

[B52] Rozas J. Ferrer-Mata A. Sánchez-DelBarrio J. C. Guirao-Rico S. Librado P. Ramos-Onsins S. E. (2017). DnaSP 6: DNA sequence polymorphism analysis of large data sets. *Mol. Biol. Evol.* 34 3299–3302. 10.1093/molbev/msx248 29029172

[B53] Rozewicki J. Li S. Amada K. M. Standley D. M. Katoh K. (2019). MAFFT-DASH: integrated protein sequence and structural alignment. *Nucleic Acids Res.* 47 W5–W10. 10.1093/nar/gkz342 31062021 PMC6602451

[B54] Samuk K. Noor M. A. F. (2022). Gene flow biases population genetic inference of recombination rate. *G3 (Bethesda)* 12:jkac236. 10.1093/g3journal/jkac236 36103705 PMC9635666

[B55] Schmidt D. J. Pickett B. E. Camacho D. Comach G. Xhaja K. Lennon N. J. (2011). A phylogenetic analysis using full-length viral genomes of South American dengue serotype 3 in consecutive Venezuelan outbreaks reveals a novel NS5 mutation. *Infect. Genet. Evol.* 11 2011–2019. 10.1016/j.meegid.2011.09.010 21964598 PMC3565618

[B56] Schoch C. L. Ciufo S. Domrachev M. Hotton C. L. Kannan S. Khovanskaya R. (2020). NCBI Taxonomy: a comprehensive update on curation, resources and tools. *Database (Oxford)* 2020:baaa062. 10.1093/database/baaa062 32761142 PMC7408187

[B57] Siddell S. G. Smith D. B. Adriaenssens E. Alfenas-Zerbini P. Dutilh B. E. Garcia M. L. (2023). Virus taxonomy and the role of the International Committee on Taxonomy of Viruses (ICTV). *J. Gen. Virol.* 104:001840. 10.1099/jgv.0.001840 37141106 PMC10227694

[B58] Stica C. J. Barrero R. A. Murray R. Z. Devine G. J. Phillips M. J. Frentiu F. D. (2022). Global evolutionary history and dynamics of dengue viruses inferred from whole genome sequences. *Viruses* 14:703. 10.3390/v14040703 35458433 PMC9030598

[B59] Suchard M. A. Lemey P. Baele G. Ayres D. L. Drummond A. J. Rambaut A. (2018). Bayesian phylogenetic and phylodynamic data integration using BEAST 1.10. *Virus Evol.* 4:vey016. 10.1093/ve/vey016 29942656 PMC6007674

[B60] Tamura K. Stecher G. Kumar S. (2021). MEGA11: molecular evolutionary genetics analysis version 11. *Mol. Biol. Evol.* 38 3022–3027. 10.1093/molbev/msab120 33892491 PMC8233496

[B61] Tanudisastro H. A. Deveson I. W. Dashnow H. MacArthur D. G. (2024). Sequencing and characterizing short tandem repeats in the human genome. *Nat. Rev. Genet.* 25 460–475. 10.1038/s41576-024-00692-3 38366034

[B62] Torrens-Fontanals M. Peralta-García A. Talarico C. Guixà-González R. Giorgino T. Selent J. (2022). SCoV2-MD: a database for the dynamics of the SARS-CoV-2 proteome and variant impact predictions. *Nucleic Acids Res.* 50 D858–D866. 10.1093/nar/gkab977 34761257 PMC8689960

[B63] Villar L. Dayan G. H. Arredondo-García J. L. Rivera D. M. Cunha R. Deseda C. (2015). Efficacy of a tetravalent dengue vaccine in children in Latin America. *N. Engl. J. Med.* 372 113–123. 10.1056/NEJMoa1411037 25365753

[B64] Vogels C. B. F. Hill V. Breban M. I. Chaguza C. Paul L. M. Sodeinde A. (2024). DengueSeq: a pan-serotype whole genome amplicon sequencing protocol for dengue virus. *BMC Genom*. 25:433. 10.1186/s12864-024-10350-x 38693476 PMC11062901

[B65] Waman V. P. Kolekar P. Ramtirthkar M. R. Kale M. M. Kulkarni-Kale U. (2016). Analysis of genotype diversity and evolution of dengue virus serotype 2 using complete genomes. *PeerJ* 4:e2326. 10.7717/peerj.2326 27635316 PMC5012332

[B66] Wang W. Shi B. He C. Wu S. Zhu L. Jiang J. (2023). Euclidean distance-based Raman spectroscopy (EDRS) for the prognosis analysis of gastric cancer: a solution to tumor heterogeneity. *Spectrochim. Acta A. Mol. Biomol. Spectrosc.* 288:122163. 10.1016/j.saa.2022.122163 36462319

[B67] Weber M. N. Streck A. F. Silveira S. Mósena A. C. S. da Silva M. S. Canal C. W. (2015). Homologous recombination in *Pestiviruses*: identification of three putative novel events between different subtypes/genogroups. *Infect. Genet. Evol.* 30 219–224. 10.1016/j.meegid.2014.12.032 25562124

[B68] Weger-Lucarelli J. Garcia S. M. Rückert C. Byas A. O’Connor S. L. Aliota M. T. (2018). Using barcoded Zika virus to assess virus population structure *In Vitro* and in *Aedes aegypti* mosquitoes. *Virology* 521 138–148. 10.1016/j.virol.2018.06.004 29935423 PMC6309320

[B69] Welch J. J. Eyre-Walker A. Waxman D. (2008). Divergence and polymorphism under the nearly neutral theory of molecular evolution. *J. Mol. Evol.* 67 418–426. 10.1007/s00239-008-9146-9 18818860

[B70] World Health Organization (2023). *Disease Outbreak News; Dengue - Global situation.* Geneva: World Health Organization.

[B71] Xia X. (2018). DAMBE7: new and improved tools for data analysis in molecular biology and evolution. *Mol. Biol. Evol.* 35 1550–1552. 10.1093/molbev/msy073 29669107 PMC5967572

[B72] Ye J. Coulouris G. Zaretskaya I. Cutcutache I. Rozen S. Madden T. L. (2012). Primer-BLAST: a tool to design target-specific primers for polymerase chain reaction. *BMC Bioinform.* 13:134. 10.1186/1471-2105-13-134 22708584 PMC3412702

[B73] You C. Jiang S. Ding Y. Ye S. Zou X. Zhang H. (2024). RNA barcode segments for SARS-CoV-2 identification from HCoVs and SARSr-CoV-2 lineages. *Virol. Sin.* 39 156–168. 10.1016/j.virs.2024.01.006 38253258 PMC10877444

[B74] Yu S. (2023). Comparison of clinical characteristics and peripheral blood tests of COVID-19 and influenza B patients. *Am. J. Trop. Med. Hyg.* 108 1028–1030. 10.4269/ajtmh.22-0620 36913928 PMC10160907

[B75] Zhao S. L. Liang C. Y. Hong J. J. Peng H. Y. (2003). Genomic sequence analyses of segments 1 to 6 of *Dendrolimus* punctatus cytoplasmic polyhedrosis virus. *Arch. Virol.* 148 1357–1368. 10.1007/s00705-003-0103-z 12827465

[B76] Zhao S. Guo Y. Sheng Q. Shyr Y. (2014). Advanced heat map and clustering analysis using heatmap3. *Biomed. Res. Int.* 2014:986048. 10.1155/2014/986048 25143956 PMC4124803

[B77] Zhuang X. Zhao Z. Feng X. Lui G. C. Y. Chan D. Lee S. S. (2023). Integrating magnetic-bead-based sample extraction and molecular barcoding for the one-step pooled RT-qPCR assay of viral pathogens without retesting. *Anal. Chem.* 95 6182–6190. 10.1021/acs.analchem.3c00885 37005794 PMC10100384

[B78] Zimmerman M. G. Quicke K. M. O’Neal J. T. Arora N. Machiah D. Priyamvada L. (2018). Cross-reactive dengue virus antibodies augment Zika virus infection of human placental macrophages. *Cell Host Microbe* 24 731–742. 10.1016/j.chom.2018.10.008 30439342 PMC6394860

