# Supplementary figures and images for: Deciphering dengue: novel RNA barcoding segments for enhanced serotype-specific identification and global surveillance of dengue viruses
Source: Front Microbiol. 2024 Dec 23;15:1474406. doi: 10.3389/fmicb.2024.1474406 (PMC11701003; doi:10.3389/fmicb.2024.1474406)

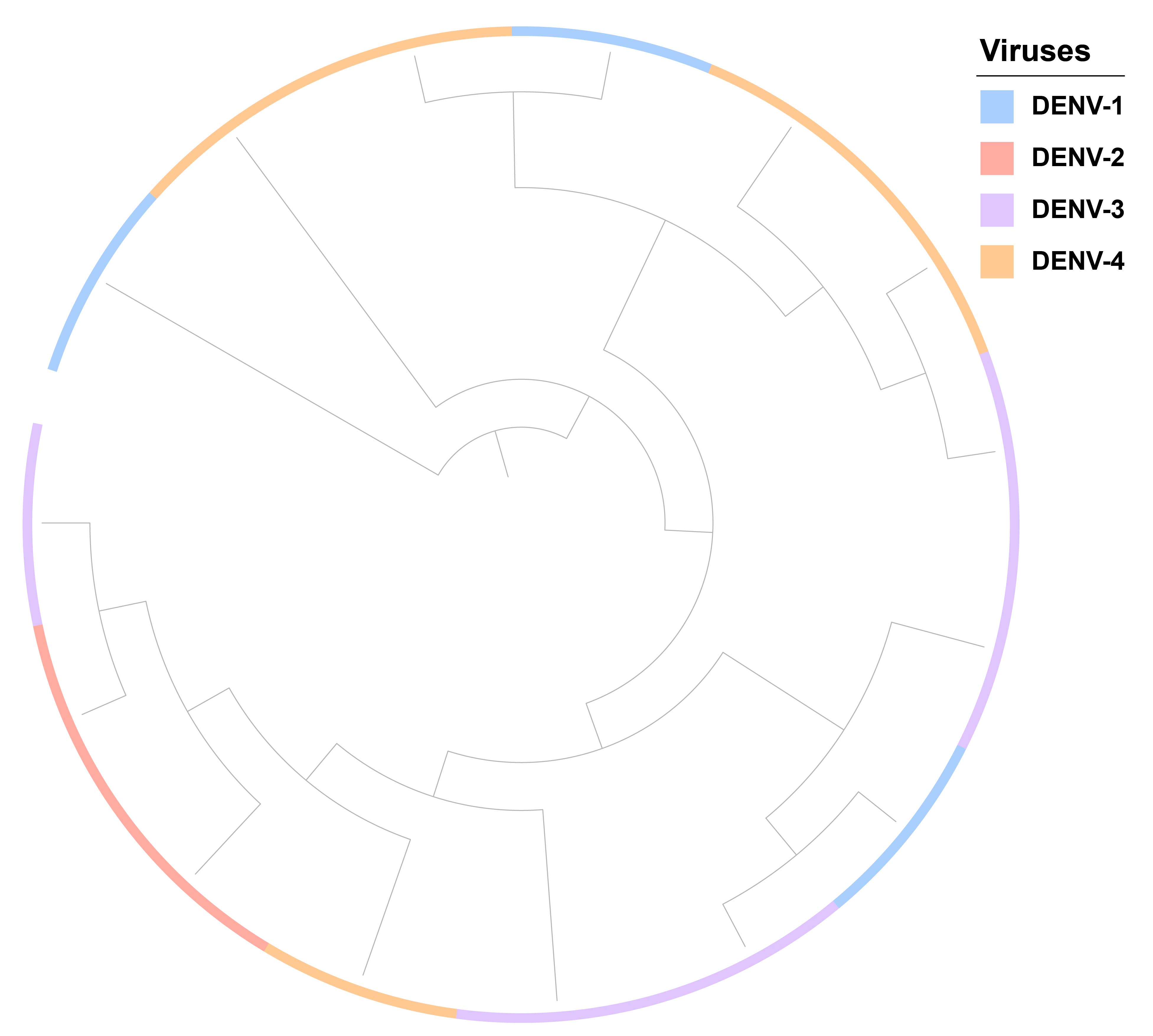

Supplement: Supplementary Figure 1 — The Bayesian phylogenetic tree of TRS. [file Data_Sheet_1.zip › Supplementary Figure S1.jpg]
